# A wide-detector CT protocol for one-stop cardio-cerebrovascular angiography: a technical feasibility study comparing an integrated protocol with conventional two-scan approach

**DOI:** 10.3389/fradi.2026.1887780

**Published:** 2026-09-01

**Authors:** Jie Yan, Feiyan Wang, Shuixian Huang, Yunhui Chen, Yan Long, Bing Ge, Xiao He

**Affiliations:** 1 Department of Radiology, Gejiu People’s Hospital, Gejiu, Yunnan, China; 2 Canon Medical Systems (CHINA) Co., LTD, Beijing, China

**Keywords:** cardiovascular, cerebrovascular, computed tomography angiography, one-stop, variable helical pitch

## Abstract

**Objective:**

To evaluate the clinical utility of a wide-detector CT protocol with variable helical pitch (vHP) for one-stop cardiovascular and cerebrovascular CT angiography.

**Methods:**

This prospective randomized study enrolled 61 patients (25 in the Group A and 36 in the Group B) with suspected cardiovascular and cerebrovascular diseases. Group A underwent the integrated one-stop CTA protocol (which incorporates vHP technology), whereas Group B underwent conventional separate scans (coronary CTA and head-neck CTA). Both groups used automated bolus-tracking triggering. Comparisons included effective scanning time, total operating time, contrast medium volume, radiation dose (dose-length product, DLP), and subjective and objective image quality. Normality was assessed using the Shapiro–Wilk test; continuous variables were compared using independent samples t-test or Mann–Whitney U test as appropriate. The false discovery rate method was applied to correct for multiple comparisons, and analysis of covariance was performed to adjust for potential confounders.

**Results:**

The integrated protocol group (Group A) demonstrated significantly higher contrast-to-noise ratio (CNR) in the coronary arteries and major aortic branches(all FDR-corrected *P* < 0.001). In the neck region, Group A showed significantly higher SNR in the common carotid arteries (FDR-corrected *P* < 0.001 for RCCA and *P* = 0.0044 for LCCA), whereas Group B demonstrated better CNR in the right internal carotid artery (FDR-corrected *P* < 0.001) and lower image noise in several vessels (FDR-corrected *P* < 0.05 for RICA SD and LVA SD). Notably, ANCOVA models for SNR exhibited poor fit with adjusted R^2^ values frequently near zero or negative, warranting cautious interpretation; the primary evidence for protocol-related image quality improvement is therefore based on the robust CNR findings. ANCOVA confirmed that these intergroup differences remained significant after adjustment (all adjusted *P* < 0.05 for key CNR/SNR comparisons), indicating protocol-related rather than baseline-driven advantages. Subjective image quality was excellent in both groups (median 4, IQR 4–4), with no significant intergroup differences. DLP was higher in Group A (909.00 vs. 694.50 mGy·cm, *P* < 0.001), but effective scanning time was significantly shorter (8.00 vs. 19.00 s, 57.9% reduction, *P* < 0.001), and total operating time was markedly reduced (480.00 vs. 1020.00 s, 52.9% reduction, *P* < 0.001). Contrast medium volume was halved in Group A (50–60 vs. 100–120 mL).

**Conclusion:**

The integrated wide-detector CT protocol evaluated in this study (which incorporates variable helical pitch as a key enabling component) is technically feasible and offers workflow advantages over conventional scanning, including shorter scan time, reduced contrast volume, and maintained image quality, with a somewhat higher radiation dose. However, lacking an independent reference standard, its diagnostic accuracy for detecting significant stenosis remains unproven. Further validation with invasive angiography is needed before routine clinical use. However, because the integrated protocol differs from the conventional protocol in multiple parameters simultaneously (detector configuration, rotation speed, pitch, and trigger threshold), the individual contribution of variable helical pitch cannot be isolated from these combined hardware and protocol differences.

## 1 Introduction

Cardiovascular and cerebrovascular diseases (CCVD), including stroke and coronary heart disease, are leading causes of mortality and disability worldwide. Atherosclerosis is a primary contributing factor to these conditions (1, 2). As the disease progresses, it leads to luminal narrowing or plaque rupture, resulting in embolism that affects the cerebral and carotid arteries and ultimately causes ischemic stroke. When the coronary arteries are involved, coronary heart disease ensues. These conditions frequently coexist and are referred to as cerebrocardiovascular comorbidities (3). With the continued aging of the population in China, the incidence of these diseases has risen annually, while the age at onset has progressively decreased, posing a serious threat to public health (4). Studies have shown that approximately 66.8% of patients with ischemic stroke have concomitant coronary atherosclerosis, whereas around 20% of individuals with coronary heart disease present with head and neck atherosclerosis (5, 6). Patients with comorbidities affecting both the brain and the cardiovascular system have a poor prognosis, as reflected by significantly higher 5-year mortality rates compared with those with ischemic stroke alone (25.3% vs. 18.5%) or coronary heart disease alone (13.1% vs. 9.2%) (7, 8).Consequently, a comprehensive and accurate assessment of atherosclerosis in both the cardiovascular and cerebrovascular arteries, as well as their interrelationship, is essential. Early intervention represents a key strategy for improving patient outcomes and reducing healthcare costs.

CT angiography (CTA) is increasingly utilized in clinical practice for evaluating cardiovascular and cerebrovascular diseases. This technique offers a comprehensive view of these systems, effectively delineating anatomical structures and lesion sites within the vessels. In contrast, traditional imaging of the cardiovascular and cerebrovascular systems typically necessitates two separate scanning sessions, which require a substantial volume of contrast agent, extended procedure times, and complex operational protocols. These factors may expose patients to excessive radiation—a concern that applies across all age groups, particularly in younger patients with longer life expectancy under the ALARA principle—and elderly patients may face additional challenges due to difficulty in cooperating and often compromised health status. Recently, numerous researchers have explored the feasibility of one-stop imaging that integrates cardiovascular and cerebrovascular scanning. The advantages of this approach,including simplicity, speed, non-invasiveness, reduced contrast agent usage, and increased image quality have contributed to its widespread adoption (9). Consequently, this study aimed to evaluate the clinical utility of an integrated one-stop cardiovascular and cerebrovascular CTA protocol incorporating vHP technology on a 320-slice wide-detector CT system, in comparison with the conventional two-scan approach.

## 2 Materials and methods

### 2.1 Research subjects

**Figure 1:**
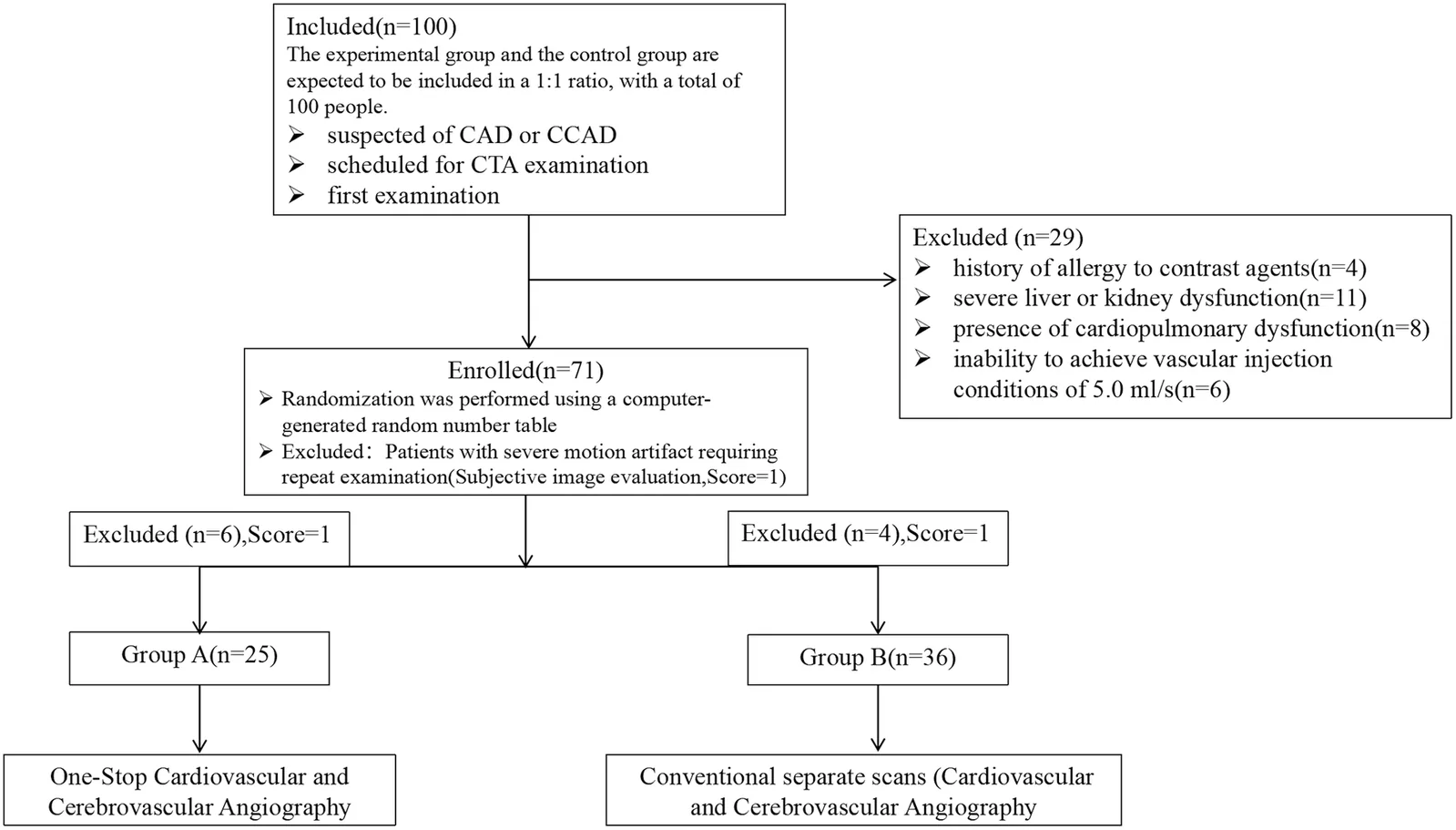
Flowchart illustrating the patient enrollment and study design.

A total of 100 patients with clinically suspected cardiovascular and cerebrovascular diseases, scheduled for head-and-neck CTA and coronary CTA examinations, were initially screened between April 25, 2024 and March 31, 2025. Of these, 29 were excluded based on predefined criteria. The remaining 71 patients were randomly assigned using a computer-generated random number table to either Group A (integrated one-stop protocol, *n* = 31) or Group B (conventional two-scan protocol, *n* = 40). Randomization was performed using a computer-generated random number table created with R version 4.2.2 (R Foundation for Statistical Computing, Vienna, Austria). The allocation sequence was generated prior to patient enrollment, and assignments were concealed using sequentially numbered, opaque, sealed envelopes. Owing to the nature of the scan protocols, the operators performing the scans were not blinded to group assignment, as the vHP and conventional protocols require different scanning modes and parameter settings. However, the two senior radiologists who independently assessed image quality were fully blinded to the patients’ group allocation, and all image evaluations were performed on anonymized datasets. After scanning, 10 patients were excluded from final analysis due to severe motion artifacts resulting in nondiagnostic images (score = 1): 6 in Group A (19.4%) and 4 in Group B (10.0%). The final analytic cohort comprised 61 patients (Group A, *n* = 25; Group B, *n* = 36), with ages ranging from 37 to 72 years (mean age 56.6 years) and a male-to-female ratio of 30:31. Baseline demographic and clinical characteristics of the two groups are summarized in Table 1. A detailed patient flowchart is presented in Figure 1.

**Table 1:** Patient and baseline characteristics by group.

Variable	Group A	Group B	t/U/*χ*²	*P* value
Age(years)	53.56 ± 10.84	58.67 ± 8.96	t=−1.94	0.059
Gender	15/25 (60.0%)	16/36 (44.4%)	χ²=0.87	0.350
SBP(mmHg)^a^	112.00 (98.00–130.00)	126.00 (119.50–150.00)	U=276	0.011＊
DBP(mmHg)^a^	80.00 (72.00–88.00)	80.00 (72.75–90.00)	U=402	0.484
HR(beats/min)	75.48 ± 8.86	76.50 ± 11.50	t=−0.39	0.697
Weight(kg)^a^	69.00 (66.00–71.00)	70.50 (69.00–74.25)	U=335	0.092
Height(m)^a^	1.67 (1.64–1.69)	1.69 (1.67–1.70)	U=340	0.103
BMI(kg/m2)^a^	24.91 (24.22–25.46)	25.00 (24.34–25.85)	U=380	0.311
Smoking history(yes)	15/25 (60.0%)	16/36 (44.4%)	χ²=0.87	0.350
Hypertension(yes)	13/25 (52.0%)	21/36 (58.3%)	χ²=0.05	0.820
Diabetes(yes)	4/25 (16.0%)	13/36 (36.1%)	χ²=2.05	0.152
Hyperlipidemia(yes)	8/25 (32.0%)	13/36 (36.1%)	χ²=0.00	0.953

Data are presented as mean ± SD, median (IQR), or n/N (%). SBP, systolic blood pressure; DBP, diastolic blood pressure; BMI, body mass index.

a Non-normally distributed variables are presented as median (interquartile range).

Inclusion criteria included: ① absence of contraindications related to contrast agents; ② individuals identified as having high clinical suspicion for cardiovascular and/or cerebrovascular diseases, defined by the presence of at least two of the following: (a) typical clinical symptoms (chest pain, palpitations, dizziness, or transient ischemic attack); (b) confirmed history of hypertension, diabetes, or hyperlipidemia; (c) long-term smoking history (>10 pack-years); or (d) abnormal preliminary screening (abnormal electrocardiogram or carotid plaque detected by ultrasound); ③ completeness of image data, free from severe motion artifacts that would render the images nondiagnostic; ④ ability to coordinate breathing, maintain a regular heart rhythm, and sustain a heart rate of less than 100 beats per minute; ⑤ awareness of and voluntary signing of the CTA informed consent form.

Exclusion criteria included: ① history of allergy to contrast agents and severe liver or kidney dysfunction; ② presence of cardiopulmonary dysfunction; ③ inability to achieve vascular injection conditions of 5.0 mL/s; ④ presence of arrhythmia or a heart rate exceeding 100 beats per minute.

To assess whether the exclusion of 10 patients due to severe motion artifacts introduced systematic bias, we compared baseline characteristics between the excluded and included patients (Supplementary Table 1). The excluded patients were significantly older than the included cohort (70.70 ± 7.94 vs. 56.57 ± 10.01 years, *P* < 0.001), while no significant differences were observed in sex, BMI, heart rate, blood pressure, or comorbidities (all *P* > 0.05). This suggests that the excluded patients were predominantly older individuals with potentially greater susceptibility to motion artifacts, consistent with the higher proportion of nondiagnostic images in Group A (19.4%) relative to Group B (10.0%). This higher exclusion rate in the integrated protocol group may reflect greater susceptibility of the small-pitch helical acquisition to motion artifacts, particularly in older patients, although operator experience and patient-selection factors cannot be excluded. Of note, the nondiagnostic rate in Group A appeared higher during the initial phase of the study, which may suggest a learning curve effect; however, formal time-series validation is not available due to the limited sample size and the absence of monthly or quarterly exclusion rate breakdowns.

### 2.2 Equipment and scanning protocol

#### 2.2.1 Equipment

Head and neck CT angiography (CTA) and coronary CTA were performed using a Canon 320-slice CT scanner (Aquilion ONE pure VISION). A high-pressure injector (Ulrich Medical, INJECT) was used for contrast material administration.

#### 2.2.2 Scanning protocol

**Figure 2:**
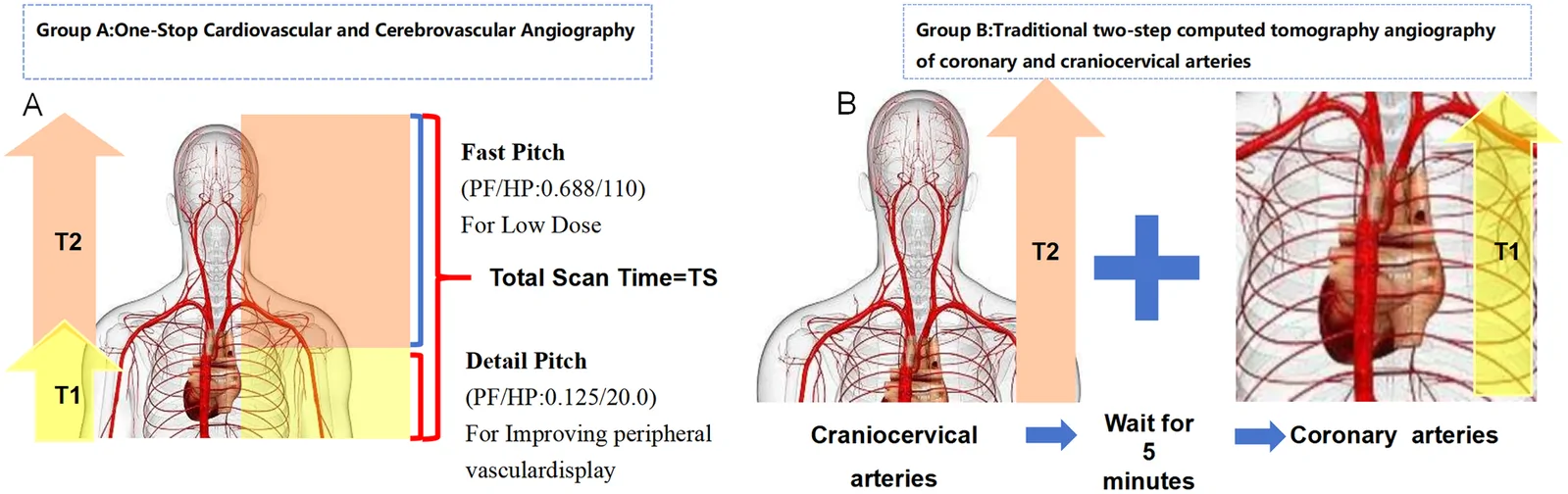
Scanning scheme diagram. (**A**) Group A (Under the integrated one-stop CTA protocol which incorporates vHP technology). T1 represents the scan time of the coronary arteries; T2 represents the scan time of the craniocervical arteries; and TS is the total scanning time. During the scanning process, the orange area adopts the fastpitch scanning mode, while the yellow area adopts the detailpitch scanning mode. (**B**) Group B (Traditional twostep computed tomography angiography of coronary and craniocervical arteries). The craniocervical arteries are scanned first (T2), followed by a 5 minute waiting period, after which the coronary arteries are scanned (T1). The total examination time is the sum of T1, T2, and the 5 minute interval.

Before scanning, patients in both groups received breathing instructions. A 20-gauge high-pressure indwelling cannula was placed in the right antecubital vein. Patients were positioned supine, with the head placed first, and electrocardiogram leads were properly connected to obtain reliable gating signals. The acquisition window for coronary artery imaging was set to 30%–80% of the R-R interval for all patients. Prior to contrast injection, both groups underwent coronary artery calcium scoring and plain neck CT scans using identical parameters. Both groups received a contrast agent dose of 1 mL/kg injected at a rate of 5 mL/s, using iodixanol (320 mg I/mL). As Group A underwent a single one-stop scan, its total contrast volume was approximately half that of Group B, which required two separate scans (coronary and head-neck CTA) with the same per-kilogram dose. Immediately after contrast injection, 40 mL of normal saline was administered at the same injection rate (Figure 2).

##### 2.2.2.1 Experimental group (group A)

A One-Stop Cardiovascular and Cerebrovascular Angiography was employed for the assessment of cardiovascular and cerebrovascular diseases. The patient's upper limbs were positioned naturally along the sides of the body. Under the integrated one-stop CTA protocol (which incorporates vHP technology), vascular imaging was performed in two distinct pitch modes. covering from the base of the heart to the cranial apex. In the first segment, extending from the cardiac base to the tracheal protrusion, the pitch factor (PF) and helical pitch (HP) were set to 0.125 and 20.0, respectively, with electrocardiogram (ECG) gating activated. The second segment, contiguous with the upper boundary of the first, was scanned from that point to the cranial apex. In this segment, the PF and HP were adjusted to 0.688 and 110.0, respectively, with ECG gating deactivated and the scanning direction oriented from foot to head. The scanning parameters included a tube voltage of 100 kV, automatic milliampere (mA) modulation, a tube rotation time of 0.275 s per revolution, a detector collimation width of 0.5 mm × 160, a matrix of 512 × 512, and a field of view (FOV) limited to 250 mm. Bolus tracking with automatic trigger technology was used, with the region of interest (ROI) placed on the descending aorta at the level of the start of the scan (cardiac base). The trigger threshold was set at 200 Hounsfield units (HU).

##### 2.2.2.2 Control group (group B)

Traditional two-step computed tomography angiography of coronary and craniocervical arteries. Initially, CTA angiography of the head and neck was conducted, followed by the injection of a contrast agent after a 5-minute interval for coronary CTA angiography. During the head and neck CTA scan, both upper limbs were positioned alongside the body. The scan extended from the tracheal protrusion to the top of the skull, with the feet oriented towards the head. Automatic trigger technology utilizing group injection tracking was implemented. The region of interest (ROI) trigger was established at the descending aorta at the scan's onset, with a trigger threshold set at 200 HU. The tube voltage was maintained at 100 kV, with automatic milliampere (mA) settings, a tube rotation speed of 0.5 s per revolution, and a collimation width of 0.5 mm × 100. The pitch factor/helix pitch (PF/HP) was 0.810/81.0, the matrix size was 512 × 512, and the field of view (FOV) was restricted to below 250;Following the completion of the CTA scan of the head and neck, a waiting period of 5 minutes was observed before raising both upper limbs and initiating the coronary CTA scan. Volume data were collected without any movement of the bed. The region of interest (ROI) was positioned in the descending aorta at the midpoint of the scan range. The ROI trigger threshold was set at 280 HU, with a tube voltage of 100 kV, automatic mA, and a tube rotation speed of 0.275 s per revolution. The collimation width was 0.5 mm × Auto, the matrix was 512 × 512, and the field of view (FOV) was maintained at less than 250.

For Group B, the pitch of the coronary CTA was adjusted according to the patient‘s actual heart rate during scanning, following the standard institutional protocol. In contrast, the first-segment pitch in the integrated one-stop CTA protocol(which incorporates vHP technology) (Group A) was fixed, as the vHP mode requires a consistent pitch to enable seamless transition between the ECG-gated and non-gated phases within a single acquisition. Therefore, heart-rate-dependent pitch modulation was not applied in Group A.

### 2.3 Image reconstruction parameters and post-processing

The reconstruction parameters for both groups are summarized in Table 2. After scan completion, the images from both groups were transferred to a Canon Vitrea post-processing workstation. Maximum intensity projection (MIP) was applied to the target vessels using the post-processing software. In addition, multi-planar reconstruction (MPR), curved planar reformation (CPR), and volume rendering (VR) were used to evaluate the target vessels.

**Table 2:** Image reconstruction parameters used in group A and B.

Parameter	Group A	Group B	
	CTA Body	CTA Neck	CCTA Cardiac
Gating	ON	/	ON
SureCardio(Helical)	CTA/CFA Mod 30%～80%	/	Prosp CTA 30%～80%
ReconFC	FC04/43	FC04	FC43
OSR	Body	Body	Cardiac
Recon Process	AIDR 3D Standard	AIDR 3D Standard	AIDR 3D Standard
Interp	V-TCOT	V-TCOT	voIumeXact
SIice Thickness	1.0mm/0.5mm	1.0mm	0.5mm
SIice IntervaI	1.0mm/0.5mm	1.0mm	0.5mm

AIDR 3D, adaptive iterative dose reduction 3D; STD, standard; CTA, computed tomography angiography.

### 2.4 Image quality evaluation

Subjective image evaluation was conducted independently by two senior attending physicians, each possessing over 10 years of experience, who performed a double-blinded assessment of images from both groups. Any discrepancies were resolved through consensus following consultation. A four-point scoring system was employed for a comprehensive assessment of image quality. The evaluation was performed by reviewing all available reconstructions, including axial source images, multi-planar reconstruction (MPR), curved planar reformation (CPR), and volume rendering (VR) images. The evaluation criteria were established as follows: a score of 4 indicated excellent image quality, characterized by clear vascular depiction and the absence of motion artifacts; a score of 3 denoted good quality, with minimal motion artifacts that still satisfied diagnostic requirements; a score of 2 represented moderate quality, exhibiting blurred vascular wall delineation and motion artifacts while remaining diagnostically usable; and a score of 1 indicated that the image was nondiagnostic.

Objective image evaluation: All indicators were assessed on cross-sectional images.

**Figure 3:**
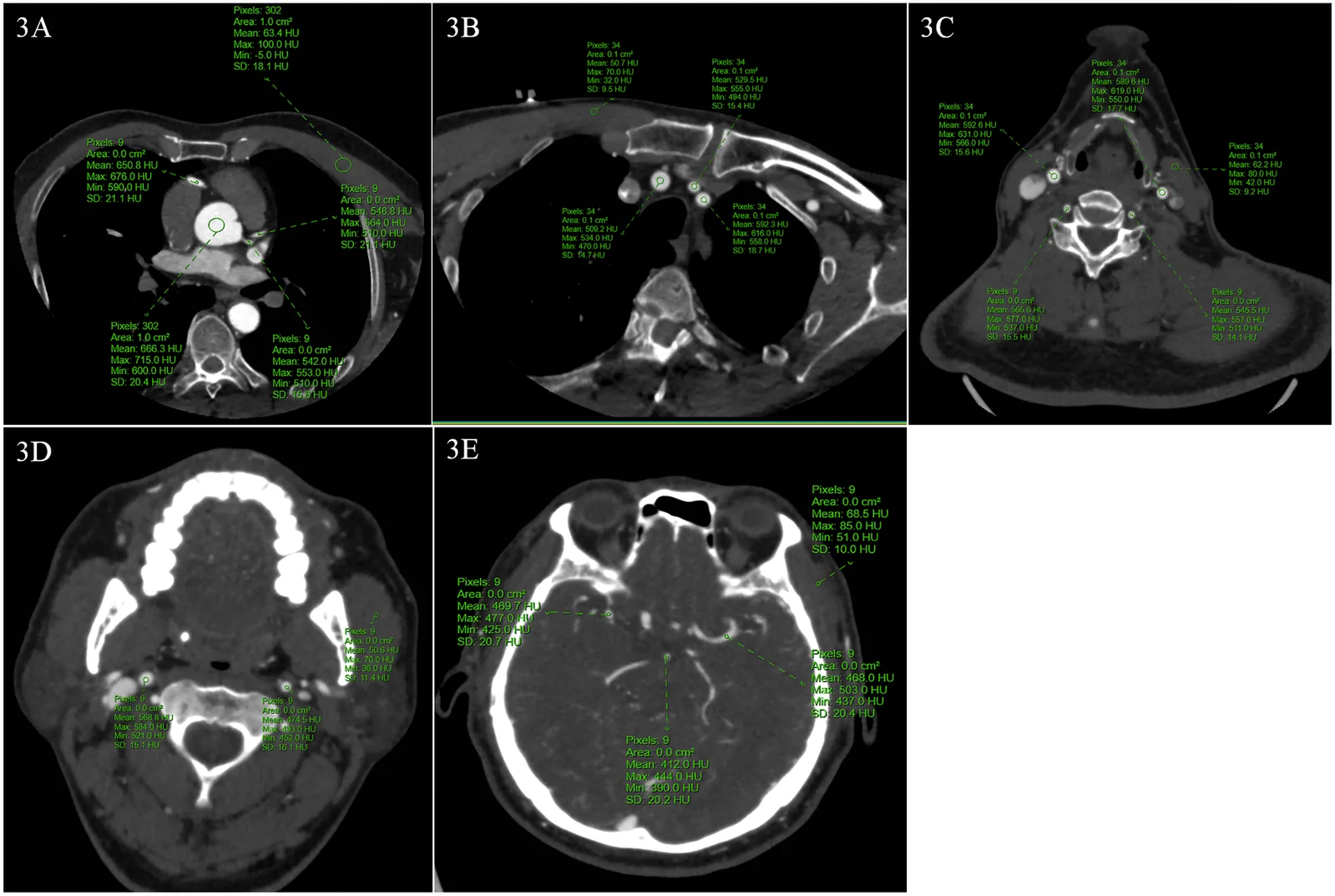
Example of objective image quality measurement. The green circles indicate the regions of interest (ROIs) for the target blood vessels and the muscles at the same anatomical level. Panels **A–E** correspond to the following locations: **A**, root level of the ascending aorta (muscle: pectoralis major); **B**, level of the three major branches of the aortic arch (muscle: pectoralis major); **C**, level of the bilateral common carotid arteries and vertebral arteries at the thyroid cartilage (muscle: sternocleidomastoid); **D**, level of the internal carotid artery at the cervical spine (muscle: sternocleidomastoid); and **E**, level of the basilar artery and the M1 segment of the bilateral middle cerebral arteries (muscle: temporalis).

(1) Evaluation of coronary artery image quality: CT values and standard deviation (SD) values were measured at the proximal openings of the ascending aorta (root and arch), as well as at the right coronary artery, left anterior descending artery, and circumflex artery. In addition, CT and SD values of the chest wall muscles were recorded at the level of the left main coronary artery originating from the aortic root. These measurements were used to calculate the signal-to-noise ratio (SNR) and contrast-to-noise ratio (CNR) for coronary computed tomography angiography (CTA) and the ascending aorta.
(2) Evaluation of head and neck artery image quality: CT and SD values of the muscles at the same levels were recorded to calculate the SNR and CNR for each head and neck vessel. The following specific muscles were used as background reference at each anatomical level: pectoralis major at the aortic root and aortic arch levels; sternocleidomastoid muscle at the thyroid cartilage level (bilateral common carotid and vertebral arteries); and temporalis muscle at the intracranial level (internal carotid artery, basilar artery, and M1 segments of the middle cerebral arteries). ROI placement was standardized across all patients to ensure measurement consistency. As illustrated in Figure 3, the region of interest (ROI) was placed to occupy approximately one-half to two-thirds of the arterial diameter, while carefully avoiding vessel margins, visible stenoses, and calcified plaques. The formulas used were as follows:
  $$CNR=\frac{\mathrm{CT}\;\mathrm{value}\;\mathrm{of}\;\mathrm{blood}\;\mathrm{vessel}\;\text{–}\;\mathrm{CT}\;\mathrm{value}\;\mathrm{of}\;\mathrm{muscle}}{\mathrm{SD}\;\mathrm{of}\;\mathrm{muscle}}$$
  $$SNR=\frac{\mathrm{CT}\;\mathrm{value}\;\mathrm{of}\;\mathrm{blood}\;\mathrm{vessel}\;}{\mathrm{SD}\;\mathrm{of}\;\mathrm{muscle}}$$

### 2.5 Radiation dose, contrast agent dosage, and examination duration

Due to the limitations of our PACS archiving system, we were unable to retrieve per-patient effective mAs data for the full cohort. Regarding CTDIvol, we obtained technical clarification from the manufacturer's engineers, which confirmed that under the vHP one-stop protocol, the system outputs a single CTDIvol value for the entire combined acquisition, whereas the conventional two-scan protocol generates separate CTDIvol readings for each scan. Because these values are calculated differently and are not directly comparable, we do not report CTDIvol data in this study. In addition, the Canon 320-slice CT system does not provide effective mAs as a discrete output parameter, as both protocols employ intelligent automatic tube current modulation. The dose–length product (DLP) was automatically recorded from the CT scanner for each patient. The total DLP for Group A was derived from the combined cardiac and craniocervical phases, whereas the total DLP for Group B was calculated as the sum of the two separate scans (coronary CTA and head–neck CTA). According to ICRP Publication 103 (10), effective dose (ED) should be calculated using region-specific k-factors for each anatomical segment. However, as segment-specific DLP values could not be retrieved retrospectively from the raw data, ED was not calculated in this study. Therefore, radiation exposure was compared between groups using total DLP only.

The dosage of the contrast agent was established at 1 mL/kg. For the experimental group, the contrast agent dosage was aligned with the one-stop imaging dosage for cardiovascular and cerebrovascular computed tomography angiography (CTA). In contrast, the control group received a dosage equivalent to the combined total of the two examinations: coronary artery CTA and head and neck CTA. The scanning times for both groups were derived from the scanning parameters of the patients.

### 2.6 Statistical analysis

All statistical analyses were performed using R version 4.2.2 (R Foundation for Statistical Computing, Vienna, Austria). The Shapiro–Wilk test was used to assess the normality of continuous variables. Variables following a normal distribution are presented as mean ± standard deviation (SD), and comparisons between groups were performed using the independent samples t-test. Variables not following a normal distribution are expressed as median with interquartile range (IQR), and the Mann–Whitney U test was applied for group comparisons. Categorical variables are reported as frequencies with percentages and were compared using the chi-square test or Fisher‘s exact test, as appropriate. To control for type I error inflation due to multiple comparisons, the false discovery rate (FDR) method was applied to all 68 objective image quality parameters, with a significance threshold set at *P* < 0.05 after correction. To further adjust for potential confounding factors, analysis of covariance (ANCOVA) was performed with age, body mass index, heart rate, and systolic blood pressure included as covariates. Adjusted least-squares means (LSMeans), between-group differences, standard errors, and 95% confidence intervals were reported. Subjective image quality was independently evaluated by two radiologists using a 4-point scoring system (1 = nondiagnostic, 2 = moderate, 3 = good, 4 = excellent). Inter-observer agreement was assessed using weighted Kappa coefficients. The distribution of subjective scores across vascular territories is presented as frequencies with percentages, and intergroup comparisons of median scores were performed using the Mann–Whitney U test. All statistical tests were two-sided, and a *P*-value < 0.05 was considered statistically significant.

## 3 Results

A total of 61 patients were prospectively enrolled and randomly assigned to either Group A (integrated one-stop protocol, *n* = 25) or Group B (conventional two-scan protocol, *n* = 36). Baseline demographic and clinical characteristics of the two groups are summarized in Table 1. The two groups were comparable with respect to age (53.56 ± 10.84 vs. 58.67 ± 8.96 years, *p* = 0.059), heart rate (75.48 ± 8.86 vs. 76.50 ± 11.50 bpm, *p* = 0.697), body mass index [24.91 [24.22–25.46] vs. 25.00 [24.34–25.85] kg/m^2^, *p* = 0.311], and sex distribution (60.0% vs. 44.4% male, *p* = 0.350). However, the control group (Group B) had a significantly higher systolic blood pressure compared with the experimental group [126.00 [119.50–150.00] vs. 112.00 [98.00–130.00] mmHg, *p* = 0.011]. No statistically significant differences were observed between the two groups in terms of diastolic blood pressure, weight, height, smoking history, hypertension, diabetes, or hyperlipidemia (all *p* > 0.05).

### 3.1 Comparison of objective image quality between groups A and B: coronary arteries, ascending aorta, and three Major supra-aortic branches

The experimental group (Group A) demonstrated significantly better results than the control group (Group B) in several objective parameters (Table 3). Specifically, Group A showed significantly higher CNR in the right coronary artery (RCA), left anterior descending artery (LAD), left circumflex artery (LCX), aortic root, aortic arch, left subclavian artery (LSA), left common carotid artery (LCCA), and brachiocephalic trunk (BCT), with FDR-corrected *P* values all < 0.001. SNR was significantly higher in Group A for the aortic root (FDR-corrected *P* = 0.018) and aortic arch (FDR-corrected *P* < 0.001); however, the SNR difference in the BCT did not reach statistical significance after FDR correction (FDR-corrected *P* = 0.091). Regarding image noise, Group A demonstrated significantly lower SD values in the LAD (FDR-corrected *P* = 0.032) and BCT (FDR-corrected *P* = 0.033), whereas the SD differences in the RCA (FDR-corrected *P* = 0.101) and LCX (FDR-corrected *P* = 0.064) were not statistically significant after FDR correction. CT values and SNR for the remaining vessels showed no statistically significant differences between the two groups after FDR adjustment (all FDR-corrected *P* > 0.05). These findings indicate that the integrated one-stop protocol provides superior contrast-to-noise performance in the coronary arteries and adjacent major cardiac vessels, primarily driven by improved CNR rather than SNR.

**Table 3:** Comparison of objective image quality between groups A and B: coronary arteries, ascending aorta, and three Major supra-aortic branches.

Vessel	Variable	Group A	Group B	Diff	95% CI	t/U/x²	Raw *P* value	FDR-corrected *P* value
RCA	CT value^a^	527.00 (516.00–551.00)	534.00 (520.50–553.50)	−7.000	[−21.000, 9.000]	U=405.50	0.518618	0.584452
	SD value	17.89 ± 1.32	18.49 ± 0.76	−0.601	[−1.195, −0.006]	t=−2.05	0.047715	0.100680
	CNR	35.15 ± 4.96	30.26 ± 3.82	4.894	[2.515, 7.272]	t=4.15	<0.001	<0.001*
	SNR	29.92 ± 3.05	29.07 ± 2.03	0.845	[−0.567, 2.256]	t=1.21	0.233382	0.322762
LAD	CT value^a^	523.00 (511.00–534.00)	525.50 (519.00–537.00)	−2.500	[−18.000, 10.000]	U=396.00	0.432351	0.501836
	SD value^a^	18.10 (17.70–18.90)	18.70 (18.40–19.02)	−0.600	[−1.000, −0.300]	U=273.00	0.009415	0.031696*
	CNR	34.64 ± 4.83	29.83 ± 3.90	4.813	[2.466, 7.160]	t=4.13	<0.001	<0.001*
	SNR	28.82 ± 2.19	28.24 ± 1.44	0.580	[−0.432, 1.592]	t=1.16	0.253283	0.329268
LCX	CT value^a^	522.00 (511.00–527.00)	523.50 (514.25–533.50)	−1.500	[−16.000, 5.512]	U=380.50	0.311321	0.367925
	SD value	18.34 ± 1.12	18.97 ± 0.90	−0.629	[−1.171, −0.088]	t=−2.34	0.023668	0.064102*
	CNR	34.24 ± 4.02	29.60 ± 3.80	4.636	[2.581, 6.691]	t=4.53	<0.001	<0.001^＊^
	SNR	28.49 ± 1.98	27.70 ± 1.44	0.794	[−0.139, 1.728]	t=1.72	0.093222	0.163769
Aorta root	CT value	544.36 ± 19.44	539.31 ± 17.70	5.054	[−4.757, 14.865]	t=1.04	0.305549	0.367925
	SD value^a^	18.20 (18.10–18.90)	18.00 (18.00–19.00)	0.200	[0.100, 0.800]	U=589.50	0.036268	0.088119
	CNR	36.18 ± 4.73	30.49 ± 3.89	5.686	[3.377, 7.994]	t=4.96	<0.001	<0.001*
	SNR^a^	30.60 (28.99–31.32)	28.85 (27.73–29.97)	1.750	[−0.060, 2.460]	U=641.50	0.005086	0.018365*
Aorta arch	CT value^a^	551.00 (531.00–566.00)	534.00 (520.50–553.50)	17.000	[−2.012, 34.000]	U=593.00	0.036603	0.1952
	SD value^a^	17.80 (17.50–18.30)	18.10 (17.70–18.52)	−0.300	[−0.700, −0.050]	U=327.50	0.073032	0.139620
	CNR	36.51 ± 5.16	30.26 ± 3.82	6.247	[3.800, 8.693]	t=5.15	<0.001	<0.001*
	SNR^a^	30.96 (29.34–32.23)	28.45 (27.62–29.70)	2.510	[0.640, 3.220]	U=709.50	<0.001	<0.001*
LSA	CT value	524.08 ± 17.28	518.69 ± 12.78	5.386	[−2.809, 13.580]	t=1.33	0.191835	0.271071
	SD value^a^	18.90 (18.10–19.80)	18.15 (17.98–18.90)	0.750	[−0.100, 1.350]	U=591.00	0.038963	0.090451
	CNR	37.37 ± 3.54	29.07 ± 3.65	8.293	[6.421, 10.165]	t=8.89	<0.001	<0.001*
	SNR^a^	27.99 (25.71–28.74)	27.90 (27.38–28.97)	0.090	[−1.900, 1.010]	U=414.50	0.607700	0.658341
LCCA	CT value^a^	516.00 (508.00–542.00)	511.50 (504.75–522.75)	4.500	[−8.000, 21.000]	U=545.50	0.163391	0.241374
	SD value^a^	18.80 (18.30–19.40)	19.00 (18.68–19.42)	−0.200	[−0.700, 0.200]	U=409.50	0.556984	0.613627
	CNR	37.56 ± 4.29	28.74 ± 3.87	8.824	[6.667, 10.982]	t=8.22	<0.001	<0.001*
	SNR^a^	27.62 (26.41–29.66)	27.15 (26.50–28.35)	0.470	[−0.930, 2.020]	U=484.50	0.617977	0.658501
BCT	CT value^a^	521.00 (511.00–527.00)	511.00 (505.50–517.25)	10.000	[−1.500, 15.512]	U=611.00	0.018345	0.051844
	SD value^a^	18.50 (17.90–18.70)	18.90 (18.40–19.02)	−0.400	[−1.000, 0.000]	U=275.50	0.010504	0.032513*
	CNR	37.25 ± 3.35	28.65 ± 3.61	8.602	[6.798, 10.406]	t=9.56	<0.001	<0.001*
	SNR	28.37 ± 3.06	26.96 ± 1.50	1.410	[0.065, 2.756]	t=2.14	0.040448	0.090659

Diff, Difference value; CI, confidence interval; FDR, false discovery rate. *A total of 68 hypothesis tests were conducted, and the FDR-adjusted significance threshold was *P*< 0.050.

a Data not following a normal distribution are expressed as median (IQR).

### 3.2 Comparison of objective image quality between groups A and B: the common carotid artery (CCA), internal carotid artery(ICA), and vertebral artery (VA)

In the neck region (Table 4), Group A demonstrated significantly higher SNR in both the left common carotid artery (LCCA) and the right common carotid artery (RCCA) at the thyroid cartilage level (FDR-corrected *P* = 0.0044 and *P* < 0.001, respectively). However, the CNR differences in these vessels did not reach statistical significance after FDR correction (LCCA: FDR-corrected *P* = 0.1376; RCCA: FDR-corrected *P* = 0.1410). Notably, while the SNR of the LCCA was significantly higher in Group A (28.78 vs. 26.50, FDR-corrected *P* = 0.0044), the CNR trended higher in Group B (38.24 ± 2.62 vs. 36.78 ± 3.25), although this difference was not statistically significant (FDR-corrected *P* = 0.1376). Similarly, for the RCCA, Group A showed significantly higher SNR (28.93 ± 2.93 vs. 26.10 ± 1.16, FDR-corrected *P* < 0.001), whereas the CNR difference did not reach statistical significance (36.83 ± 3.29 vs. 38.31 ± 2.84, FDR-corrected *P* = 0.1410).

**Table 4:** Comparison of objective image quality between groups A and B: the common carotid artery(CCA), internal carotid artery(ICA), and vertebral artery(VA).

Vessel	Variable	Group A	Group B	Diff	95% CI	t/u/x²	Raw *P* value	FDR-corrected *P* value
LCCA(The level of the thyroid cartilage)	CT value^a^	511.00 (501.00−527.00)	508.00 (498.00–519.00)	3.000	[−6.000, 18.500]	U=540.0	0.189038	0.271071
	SD value	18.34 ± 1.03	18.69 ± 0.77	−0.349	[−0.838, 0.140]	t=−1.439	0.157480	0.241374*
	CNR	36.78 ± 3.25	38.24 ± 2.62	−1.463	[−3.042, 0.116]	t=−1.867	0.068566	0.137617
	SNR	28.78 (26.83–30.52)	26.50 (25.77–27.50)	2.280	[0.559, 3.600]	U=674.5	0.001018	0.004411*
RCCA(The level of the thyroid cartilage)	CT value^a^	511.00 (497.00–527.00)	507.00 (498.75–514.75)	4.000	[−9.012, 17.000]	U=519.5	0.310968	0.367925
	SD value	18.04 ± 0.80	18.94 ± 0.54	−0.900	[−1.272, −0.529]	t=−4.908	<0.001	<0.001
	CNR	36.83 ± 3.29	38.31 ± 2.84	−1.472	[−3.104, 0.160]	t=−1.815	0.075926	0.141005
	SNR	28.93 ± 2.93	26.10 ± 1.16	2.828	[1.568, 4.088]	t=4.59	<0.001	<0.001*
LICA(Cervical vertebra level)	CT value	506.40 ± 26.06	498.33 ± 12.73	8.067	[−3.397, 19.531]	t=1.43	0.161469	0.241374
	SD value	19.28 ± 1.15	18.97 ± 0.90	0.311	[−0.243, 0.864]	t=1.13	0.263931	0.331787
	CNR	35.87 ± 3.50	37.40 ± 2.91	−1.530	[−3.244, 0.185]	t=−1.80	0.079119	0.142854
	SNR^a^	25.86 (25.02–28.34)	26.05 (25.27–26.73)	−0.190	[−0.820, 1.351]	U=451.00	0.994148	0.994148
RICA(Cervical vertebra level)	CT value	504.92 ± 18.58	521.72 ± 15.79	−16.802	[−25.965, −7.639]	t=−3.69	<0.001	0.002735*
	SD value^a^	19.50 (18.70–19.70)	18.70 (18.00–19.00)	0.800	[0.400, 1.400]	U=701.50	<0.001	0.001132*
	CNR	35.72 ± 2.81	39.53 ± 3.09	−3.804	[−5.334, −2.274]	t=−4.98	<0.001	<0.001*
	SNR^a^	25.22 (24.28–26.56)	25.75 (24.85–26.50)	−0.530	[−1.240, 0.750]	U=418.50	0.649246	0.669857
LVA (The level of the thyroid cartilage)	CT value^a^	491.00 (476.00–502.00)	487.50 (471.75–492.00)	3.500	[−8.000, 16.000]	U=546.50	0.158707	0.241374
	SD value	19.76 ± 1.10	18.97 ± 0.90	0.787	[0.249, 1.324]	t=2.95	0.005055	0.018365*
	CNR^a^	34.86 (32.22–36.32)	35.39 (33.22–38.17)	−0.525	[−3.685, 1.332]	U=340.50	0.109937	0.188051
	SNR	24.74 ± 1.91	25.28 ± 1.57	−0.544	[−1.477, 0.389]	t=−1.17	0.246645	0.327183
RVA(The level of the thyroid cartilage)	CT value^a^	488.00 (466.00–497.00)	475.00 (466.00–489.00)	13.000	[−10.000, 23.512]	U=574.00	0.069867	0.137617
	SD value	19.52 ± 1.09	19.14 ± 0.66	0.377	[−0.118, 0.873]	t=1.54	0.131469	0.213637
	CNR	34.11 ± 3.85	35.20 ± 3.06	−1.090	[−2.952, 0.772]	t=−1.18	0.244243	0.327183
	SNR^a^	24.17 (23.38–25.23)	24.75 (24.00–25.57)	−0.580	[−1.200, 0.250]	U=346.50	0.130818	0.213637

Diff, Difference value; CI, confidence interval; FDR, false discovery rate. *A total of 68 hypothesis tests were conducted, and the FDR-adjusted significance threshold was P < 0.050.

a Data not following a normal distribution are expressed as median (IQR).

Conversely, the control group (Group B) performed significantly better in several parameters at the cervical level. Group B demonstrated significantly higher CNR in the right internal carotid artery (RICA) (39.53 ± 3.09 vs. 35.72 ± 2.81, FDR-corrected *P* < 0.001), as well as significantly higher CT values in the RICA (521.72 ± 15.79 vs. 504.92 ± 18.58, FDR-corrected *P* = 0.0027). Additionally, Group B showed lower SD values (indicating less noise) in the RICA (median 18.70 vs. 19.50, FDR-corrected *P* = 0.001132) and the left vertebral artery (LVA) (18.97 ± 0.90 vs. 19.76 ± 1.10, FDR-corrected *P* = 0.0184). For the remaining vessels, CT values, CNR, and SNR showed no statistically significant differences between the two groups after FDR adjustment (all FDR-corrected *P* > 0.05).

### 3.3 Comparison of objective image quality between groups A and B: the M1 segment of the middle cerebral artery (MCA) and the basilar artery (BA)

In the intracranial region, the control group (Group B) demonstrated significantly better results than Group A for several objective parameters (Table 5). Group B showed significantly lower SD values (indicating less noise) in the left M1 segment of the middle cerebral artery (LMCA M1) (20.18 ± 0.78 vs. 19.67 ± 0.63, FDR-corrected P = 0.031696) and the basilar artery (BA) (22.83 ± 0.82 vs. 22.20 ± 0.65, FDR-corrected P = 0.009246), as well as significantly higher SNR in the BA (19.12 ± 1.19 vs. 19.84 ± 0.89, FDR-corrected P = 0.040644). In contrast, the SNR difference in the LMCA M1 did not retain statistical significance after FDR correction (median 22.89 vs. 23.80, FDR-corrected P = 0.088119). No statistically significant differences were observed in CT values or SNR for the remaining intracranial vessels after FDR adjustment (all FDR-corrected P > 0.05).

**Table 5:** Comparison of objective image quality between groups A and B: the M1 segment of the middle cerebral artery(MCA) and the basilar artery(BA).

Vessel	Variable	Group A	Group B	Diff	95% CI	t/u/x²	Raw *P* value	FDR-corrected *P* value
LMCA M1	CT value	461.08 ± 22.58	463.53 ± 16.00	−2.448	[−13.045, 8.149]	t=−0.47	0.643195	0.669857
	SD value	20.18 ± 0.78	19.67 ± 0.63	0.511	[0.130, 0.891]	t=2.70	0.009753	0.031696*
	SNR^a^	22.89 (21.68–23.83)	23.80 (23.00–24.62)	−0.910	[−2.000, 0.040]	U=305.50	0.034651	0.088119
RMCAM1	CT value^a^	454.00 (431.00–476.00)	458.50 (449.75–467.25)	−4.500	[−22.000, 14.000]	U=437.50	0.860237	0.873679
	SD value^a^	20.80 (20.20–21.20)	19.80 (19.35–20.73)	1.000	[0.200, 1.200]	U=585.00	0.048017	0.100680
	SNR	22.27 ± 2.10	22.80 ± 1.28	−0.533	[−1.489, 0.423]	t=−1.13	0.265429	0.331787
BA	CT value	436.08 ± 22.78	439.50 ± 16.11	−3.420	[−14.107, 7.267]	t=−0.65	0.521511	0.584452
	SD value	22.83 ± 0.82	22.20 ± 0.65	0.635	[0.240, 1.029]	t=3.24	0.002276	0.009246*
	SNR	19.12 ± 1.19	19.84 ± 0.89	−0.723	[−1.290, −0.156]	t=−2.57	0.013756	0.040644*

Diff, Difference value; CI, confidence interval; FDR, false discovery rate. *A total of 68 hypothesis tests were conducted, and the FDR-adjusted significance threshold was *P*< 0.050.

a Data not following a normal distribution are expressed as median (IQR).

To adjust for potential confounding factors, particularly the baseline difference in systolic blood pressure between the two groups, we performed an analysis of covariance (ANCOVA) for all objective image quality parameters, including age, body mass index, heart rate, and systolic blood pressure as covariates. The full ANCOVA results; including adjusted least-squares means, between-group differences, standard errors, 95% confidence intervals, F-values, P-values, and adjusted R^2^—are presented in Supplementary Table 2.

After adjustment, the results remained consistent with the unadjusted analyses. Group A continued to show significantly higher CNR in the RCA, LAD, LCX, aortic root, aortic arch, LSA, LCCA, and BCT, with adjusted P-values all <0.001. SNR remained significantly higher in Group A for the aortic root (adjusted *P* = 0.004), aortic arch (adjusted *P* < 0.001), LCCA at the thyroid cartilage level (adjusted *P* < 0.001), RCCA (adjusted *P* < 0.001), and BCT (adjusted *P* = 0.020). The CNR advantage in Group B for the RICA also persisted after adjustment (adjusted *P* < 0.001).

Notably, the explanatory power of the ANCOVA models, as indicated by adjusted R^2^, differed substantially between the two types of parameters. For CNR models, adjusted R^2^ values ranged from 0.183 to 0.594, indicating that the covariates explained a moderate to substantial proportion of the variance in CNR across different vascular territories (e.g., BCT CNR: adjusted R^2^ = 0.594; LSA CNR: adjusted R^2^ = 0.562; LCCA CNR: adjusted R^2^ = 0.536). This suggests that the adjusted baseline characteristics are relevant determinants of contrast-to-noise performance and that our primary CNR findings are robust to confounding. In contrast, the SNR models demonstrated consistently poor fit, with adjusted R^2^ values frequently close to zero or even negative (e.g., LICA SNR: −0.0108; RICA SNR: −0.0147; RVA SNR: −0.0128). A negative adjusted R^2^ indicates that the model with covariates explains less variance than a null model, implying that the measured baseline factors accounted for little to none of the variability in SNR. This pattern suggests that SNR is influenced by unmeasured factors; such as patient positioning, contrast injection technique, or vessel-specific anatomical variations that were not captured by our covariate set. Consequently, the adjusted SNR results derived from these poorly fitting models should be regarded as exploratory rather than confirmatory, and the primary evidence for SNR differences should be based on the unadjusted comparisons presented in Tables 3–5.

This differential model performance carries important implications for interpretation. The observation that CNR models show moderate to good explanatory power while SNR models perform poorly indicates that CNR, which reflects contrast discrimination between the vessel lumen and surrounding tissue, may be the more reliable and interpretable endpoint in this study. SNR, by contrast, appears to be more susceptible to extraneous sources of variability beyond the measured covariates, and thus SNR results should be interpreted with appropriate caution. Importantly, the poorer performance of the SNR models does not undermine the primary CNR findings, which remain robust to adjustment for baseline imbalances. Collectively, these ANCOVA findings confirm that the observed group differences in objective image quality parameters are genuinely attributable to the scanning protocol rather than being substantially influenced by baseline imbalances, particularly the difference in systolic blood pressure.

### 3.4 Comparison of radiation dose, effective scanning time and total operating time between groups A and B

Statistically significant differences were observed in dose-length product (DLP), effective scanning time, and total operating time between Group A and Group B. The DLP was significantly higher in Group A [909.00 (898.00–1000.00) mGy·cm] compared with Group B [694.50 (687.00–704.50) mGy·cm], representing a 30.8% increase (raw and FDR-corrected *P* < 0.001). To ensure that the DLP comparison was not confounded by differences in anatomical coverage, we retrieved the scan length for all patients from the DICOM tag (0018, 1302). The scan length was comparable between Group A [51.10 (48.90–52.10) cm] and Group B [50.75 (49.90–51.42) cm], with no significant intergroup difference (*P* = 0.8833; Table 6). However, the effective scanning time was significantly shorter in Group A [8.00 (7.00–9.00) s] than in Group B [19.00 (17.00–19.25) s], representing a 57.9% reduction (raw and FDR-corrected *P* < 0.001). The total operating time was also markedly shorter in Group A [480.00 (480.00–540.00) s] compared with Group B [1020.00 (900.00–1035.00) s], corresponding to a 52.9% reduction (raw and FDR-corrected *P* < 0.001). These findings indicate that although the vHP one-stop protocol resulted in a higher radiation dose (as reflected by DLP) compared with the conventional two-scan protocol, it substantially reduced both effective scanning time and total operating time. Moreover, the comparable scan lengths between the two groups confirm that the observed DLP difference reflects genuine differences in radiation exposure per unit length rather than disparities in scanned anatomical coverage.

**Table 6:** Comparison of radiation dose, effective scanning time and total operating time between groups A and B.

Variable	Group A	Group B	U	Raw *P* value	FDR-corrected *P* value
DLP	909.00 (898.00–1000.00)	694.50 (687.00–704.50)	900.00	0.0000	0.0000^*^
Scan Lengths(cm)	51.10 (48.90–52.10)	50.75 (49.90–51.42)	460.50	0.8833	-
Effective scanning time(s)	8.00 (7.00–9.00)	19.00 (17.00–19.25)	0.00	0.0000	0.0000^*^
Total operating time(s)	480.00 (480.00–540.00)	1020.00 (900.00–1035.00)	0.00	0.0000	0.0000^*^

*A total of 68 hypothesis tests were conducted, and the FDR adjusted significance threshold was *P* < 0.050.

### 3.5 Comparison of subjective image quality between groups A and B

Image quality was satisfactory for diagnostic purposes in both groups. The weighted Kappa values for inter-observer agreement across vascular territories were 0.742 (coronary arteries), 0.719 (supra-aortic branches), 0.635 (cervical arteries), and 0.658 (intracranial arteries), indicating moderate to good agreement between the two readers. Subjective image quality scores for each vascular territory, stratified by reader and group, are summarized in Table 7. For intergroup comparisons of median scores, the assessments from Reader 1 were used as the primary analysis, while those from Reader 2 were employed as a sensitivity analysis to confirm the robustness of the findings.

**Table 7:** Distribution of subjective image quality scores and intergroup comparisons of median scores across vascular territories and readers.

Vascular territory	Dr	Group	Score			Median scores (IQR)	U statistic	*P* value
			2	3	4			
Coronary Artery	Dr1	Group A	2 (8.0%)	3 (12.0%)	20 (80.0%)	4 (4–4)	448.0	0.9747
Coronary Artery	Dr1	Group B	3 (8.3%)	4 (11.1%)	29 (80.6%)			
Arch vessels	Dr1	Group A	1 (4.0%)	3 (12.0%)	21 (84.0%)	4 (4–4)	428.0	0.5904
Arch vessels	Dr1	Group B	1 (2.8%)	3 (8.3%)	32 (88.9%)			
Carotid Artery	Dr1	Group A	1 (4.0%)	1 (4.0%)	23 (92.0%)	4 (4–4)	451.0	0.9877
Carotid Artery	Dr1	Group B	1 (2.8%)	2 (5.6%)	33 (91.7%)			
Intracranial arteries	Dr1	Group A	1 (4.0%)	2 (8.0%)	22 (88.0%)	4 (4–4)	460.5	0.8025
Intracranial arteries	Dr1	Group B	3 (8.3%)	2 (5.6%)	31 (86.1%)			
Coronary Artery	Dr2	Group A	0 (0.0%)	2 (8.0%)	23 (92.0%)	4 (4–4)	501.5	0.2235
Coronary Artery	Dr2	Group B	0 (0.0%)	7 (19.4%)	29 (80.6%)			
Arch vessels	Dr2	Group A	0 (0.0%)	4 (16.0%)	21 (84.0%)	4 (4–4)	417.5	0.3958
Arch vessels	Dr2	Group B	1 (2.8%)	2 (5.6%)	33 (91.7%)			
Carotid Artery	Dr2	Group A	0 (0.0%)	3 (12.0%)	22 (88.0%)	4 (4–4)	447.5	0.9577
Carotid Artery	Dr2	Group B	1 (2.8%)	3 (8.3%)	32 (88.9%)			
Intracranial arteries	Dr2	Group A	0 (0.0%)	4 (16.0%)	21 (84.0%)	4 (4–4)	467.5	0.7086
Intracranial arteries	Dr2	Group B	1 (2.8%)	6 (16.7%)	29 (80.6%)			

## 4 Discussion

Recent advancements in CT technology have greatly enhanced the capabilities of wide-body detector systems. With a 16-cm z-axis coverage and a gantry rotation time of 0.23–0.28 s per rotation, these systems enable whole-heart coverage within a single cardiac cycle. When combined with dedicated motion artifact correction algorithms, this technology effectively suppresses vascular pulsation artifacts, thereby ensuring diagnostic image quality even in patients with elevated heart rates or arrhythmias (11–13). Consequently,one-stop imaging of the cardiovascular and cerebrovascular systems has become feasible. The clinical relevance and methodological framework of CT angiography for cardiovascular and cerebrovascular assessment are firmly established by contemporary practice guidelines and consensus documents. The 2021 AHA/ACC/ASE/CHEST/SAEM/SCCT/SCMR Guideline for the Evaluation and Diagnosis of Chest Pain elevated coronary CTA to a Class 1 recommendation with Level A evidence for patients with stable and acute chest pain, establishing it as the frontline noninvasive test for diagnosing coronary artery disease, guiding risk stratification, and informing treatment decisions (14). The SCCT 2023 Standardized Medical Terminology for Cardiac Computed Tomography provides a unified framework for image quality assessment and diagnostic reporting, ensuring consistency across clinical and research settings (15). In recent years, numerous researchers have examined the efficacy of one-stop imaging for simultaneous cardiovascular and cerebrovascular scanning. Guo et al. (16) demonstrated that one-stop cardiovascular and cerebrovascular imaging could significantly reduce both scanning time and contrast agent dosage, with image quality comparable to that achieved through two consecutive scans.

For One-Stop Cardiovascular and Cerebrovascular Angiography, the main technical challenge is that coronary artery image quality is susceptible to cardiac pulsation (13),whereas visualization of the head and neck vessels is comparatively straightforward. To address this issue, our study employed an integrated one-stop CTA protocol incorporating vHP technology on a 320-slice, 16-cm wide-detector CT system to systematically assess objective image quality, subjective image quality, examination duration, contrast agent dosage, and radiation exposure in one-stop CTA imaging for cardiovascular and cerebrovascular diseases. The findings indicated that the integrated one-stop protocol, incorporating vHP technology on a 320-slice, 16-cm wide-detector CT system, can reduce the contrast agent volume by half and significantly decrease examination time, all while maintaining image quality, corroborating existing literature. This study employed electrocardiogram-gated small-pitch scanning to ensure optimal visualization of coronary arteries. For regions outside the heart, standard pitch was utilized to facilitate conventional low-dose scanning. Both subjective and objective image quality of the major branches of coronary arteries and head and neck arteries were systematically assessed. Subjective image quality was deemed satisfactory for diagnostic purposes in both groups. For each vascular territory, the median subjective score was 4 (IQR: 4–4) for both groups, as evaluated by both readers, indicating excellent overall image quality. Intergroup comparisons conducted using the Mann–Whitney U test revealed no significant differences between Group A and Group B across any vascular territory for either reader (all *P* > 0.05), suggesting that the two protocols produced comparable subjective image quality. The weighted Kappa values for inter-observer agreement ranged from 0.635 to 0.742 across vascular territories (coronary arteries: 0.742; supra-aortic branches: 0.719; cervical arteries: 0.635; intracranial arteries: 0.658), reflecting moderate to good agreement between the two independent readers.

In recent years, increasing attention has been paid to minimizing radiation doses and reducing contrast agent volumes among radiology professionals. Lowering the contrast agent dose not only alleviates the renal burden but also reduces the incidence of post-contrast acute kidney injury (PC-AKI) and the risk of contrast agent extravasation (16–19). In our study,although the coronary artery acquisition window was set to 30%–80% of the R-R interval on the electrocardiogram for all patients in both groups, the results presented in Table 3 show that the image quality of the coronary arteries and adjacent great vessels was superior in the one-stop imaging group (Group A) compared with the control group (Group B). The superior image quality of the coronary arteries and adjacent major vessels observed in Group A warrants further mechanistic explanation. Within the integrated one-stop protocol, the cardiac phase employed a helical scan with a small pitch (PF = 0.125). This small pitch generates substantial overlapping coverage along the Z-axis, which increases the sampling density and provides redundant information for image reconstruction. As a result, image noise is effectively reduced, leading to improved SNR and CNR values. This technical advantage is reflected in our quantitative results (Table 3), where Group A demonstrated significantly higher CNR and SNR for the coronary arteries and aortic root compared to Group B, these improvements are attributable to the combined effect of multiple protocol parameters, including the small pitch, rather than to the vHP feature alone. As shown in Tables 4, 5, Group B shows higher CNR and SNR values than Group A in the bilateral internal carotid arteries, vertebral arteries, and the M1 segments of the middle cerebral arteries, as well as higher SNR in the basilar artery. However, only the CNR of the RICA (35.72 ± 2.81 vs. 39.53 ± 3.09, FDR-corrected *P* = <0.001) and the SNR of the BA (19.12 ± 1.19 vs. 19.84 ± 0.89, FDR-corrected *P* = 0.040644) reached statistical significance. This difference can be attributed to the differences in detector configuration and helical pitch between the two groups. Group A employed a helical scan with a detector configuration of 0.5 mm × 160, which was designed to eliminate the switching time between activation and deactivation of electrocardiographic gating. In contrast, Group B used a detector configuration of 0.5 mm × 100. The smaller number of detector rows in Group B reduced susceptibility to cone-beam artifacts, which contributed to the enhancement of image quality in certain vascular territories independent of pitch effects. However, this image quality improvement comes at the cost of a modestly increased radiation dose, as the overlapping acquisition inevitably exposes the scanned volume to additional radiation. The higher DLP observed in Group A[909.00 [898.00–1000.00] mGy·cm vs. 694.50 [687.00–704.50] mGy·cm in Group B] is likely related to the small-pitch helical acquisition in the cardiac segment. Therefore, the clinical adoption of the integrated one-stop protocol should weigh the diagnostic benefit of improved image quality against the trade-off of increased radiation exposure. Regarding radiation dose reporting, ICRP Publication 135 (2017) on Diagnostic Reference Levels in Medical Imaging specifies that “For CT, the DLP value used is the cumulative DLP for the entire examination”, which supports our use of cumulative DLP as the primary radiation dose metric (20). The radiation dose delivered in our integrated protocol (DLP 909 mGy·cm) is comparable to previously reported values for combined cardiocerebrovascular CT examinations, though specific DRL values for this combined indication remain to be established in future multicenter surveys. For context, published DRLs for coronary CTA alone range from 326.9 to 1300 mGy·cm (21), while reported DLP values for one-stop cardio-cerebrovascular CTA protocols vary widely depending on scan parameters and patient selection. Notably, Matsumoto et al. reported a mean DLP of 1281.6 ± 195.7 mGy·cm in a TAVI-planning CT study using VHP scanning on the same 320-detector row CT system (Aquilion ONE) with a scan length of 609.1 ± 44.1 mm and a contrast volume of 38.7 ± 8.5 mL (22). Our integrated protocol achieved a substantially lower DLP (909.00 mGy·cm) despite covering a larger anatomical range (heart to cranial apex), which may be attributable to the optimized pitch modulation and faster gantry rotation (0.275 s) in the vHP protocol. This comparison suggests that the radiation dose of our integrated protocol is within acceptable international benchmarks, although formal DRLs for this specific combined indication are needed. Furthermore, an interesting discrepancy was observed in the LCCA: while Group A demonstrated significantly higher SNR, the CNR trended higher in Group B (FDR-corrected *P* = 0.137617). This finding likely reflects the trade-off between reduced contrast volume and image noise. SNR is primarily determined by signal intensity relative to background noise; the small-pitch helical acquisition in Group A, with its overlapping Z-axis coverage, effectively reduces image noise and thus improves SNR even with lower contrast enhancement. However, CNR reflects the contrast between the vessel and surrounding tissue relative to noise. When contrast enhancement is reduced by 50% in Group A, the vessel-to-background contrast diminishes, which may offset the noise-reduction benefit and lead to a relative decline in CNR. This interpretation is consistent with recent findings in the literature. A study on vHP technology in lower extremity CTA demonstrated that contrast volume reduction of 12.7% can be achieved while maintaining overall image quality, though the trade-off between contrast dose and objective parameters such as CNR warrants attention (23). Furthermore,a study on vHP technology in “one-stop” CTA scanning of carotid and coronary arteries demonstrated that vHP one-stop scanning significantly reduced contrast agent dosage by 40.2% while maintaining overall image quality, although the authors also observed significant differences in SNR and CNR between groups (24). Shin et al. (25) compared VHP vs. VFH protocols using wide-detector CT for aorta and coronary evaluation. VHP yielded superior thoracic aorta attenuation, CNR, and SNR (all *P* < 0.001), with diagnostic quality maintained. Zhao et al. (26) compared different scanning sequences in one-stop coronary and head-and-neck CTA using a wide-detector system and found that while one-stop CTA could provide diagnostic images, objective parameters such as CNR varied depending on the scanning protocol. Zhang et al.applied deep learning-based reconstruction combined with segmented contrast injection in one-stop cardio-cerebrovascular CTA and demonstrated that contrast dose reduction of over 40% could be achieved without compromising image quality, though CNR and SNR responses varied across different vascular territories (19). Nevertheless, both groups in our study maintained CNR values well above diagnostic thresholds, and subjective image quality scores remained satisfactory (≥2 points) in all patients. These findings suggest that the 50% contrast reduction achieved with the integrated one-stop protocol is clinically acceptable, although careful attention should be paid to CNR in neck vessels when further contrast optimization is pursued.

In addition to the integrated approach evaluated in our study (which incorporates vHP technology), dual-source CT with high-pitch (Flash) technology represents another mainstream strategy for one-stop cardiocerebrovascular CTA. Wang et al. (27) recently evaluated a low-kV, low-contrast medium one-stop dual-source high-pitch integrated coronary-carotid-cerebral-aortic CTA protocol in 211 non-obese patients and reported that DLP and effective radiation dose were reduced by 51%, while contrast medium volume and iodine uptake were reduced by 13%, compared with a routine-dose protocol, with SNR and CNR values close to or slightly higher than those of the routine-dose group. Sun et al. (28) demonstrated that dual-source CT with a high-pitch spiral acquisition protocol for combined coronary and carotid-cerebrovascular CTA provides good image quality and enables comprehensive evaluation of atherosclerotic burden. Liu et al. (29) further validated the feasibility of free-breathing, non-gated, high-pitch heart-to-brain CTA using third-generation dual-source CT in patients with acute ischemic stroke, achieving a mean DLP of 129.1 ± 30.5 mGy·cm with diagnostic image quality for both cardiac chambers and carotid arteries. These findings demonstrate that dual-source high-pitch scanning can achieve substantial dose reduction while maintaining diagnostic image quality, particularly in patients with low and stable heart rates, in whom the ultra-fast acquisition (approximately 0.25 s) effectively freezes cardiac motion. However, the two technological approaches differ in several important respects. High-pitch dual-source CT achieves its ultra-low radiation dose through a very high pitch factor (up to 3.4), which requires both a stable sinus rhythm and a low heart rate (typically < 65 bpm) to avoid motion artifacts. In contrast, the integrated approach evaluated in our study, implemented on a 16-cm wide-detector CT platform with a 0.275-s gantry rotation time and incorporating vHP technology, employs a small pitch (0.125) during the ECG-gated cardiac phase to ensure optimal coronary visualization. This design enables seamless transition between ECG-gated and non-gated phases within a single acquisition, making the integrated protocol a promising candidate for future investigation in patients with higher heart rates or mild arrhythmias, a hypothesis that remains to be tested in dedicated studies. The trade-off is a 30.8% higher radiation dose [DLP: 909.00 [898.00–1000.00] mGy·cm in our integrated group vs. 694.50 [687.00–704.50] mGy·cm in the conventional group], which reflects the deliberate prioritization of image quality and broader patient applicability over dose minimization. Thus, while both approaches offer workflow advantages over conventional two-scan imaging, their optimal clinical applications differ: high-pitch dual-source CT may be preferred for low-risk patients with regular, slow rhythms in whom radiation dose is the primary concern, whereas the integrated wide-detector protocol (which incorporates vHP technology) may offer greater flexibility for patients with more challenging cardiac conditions. Future head-to-head comparative studies are warranted to further delineate the relative merits of these two strategies in different patient populations.

Previous research has demonstrated that one-stop imaging of cardiovascular and cerebrovascular diseases can significantly reduce examination time, thereby facilitating timely treatment for critically ill patients. The findings of Guo et al. (16) combined CTA significantly reduced the operational time by 21.5% (186.6 ± 60.6 vs. 237.7 ± 57.9 s,*p* < 0.001), and the contrast medium by 21.8% (74.5 ± 8.0 vs. 95.3 ± 14.5 mL, *p* < 0.001). In contrast, the effective scanning time in Group A was 8.00 (7.00–9.00) seconds, compared with 19.00 (17.00–19.25) seconds in Group B, representing a 57.9% reduction,notably, we also estimated the total operating time—including patient positioning, contrast injection, and scan preparation—which was 480.00 (480.00–540.00) seconds for Group A, compared with 1020.00 (900.00–1035.00) seconds for Group B, corresponding to a 52.9% reduction. the iodine contrast agent dosage in Group A was limited to 50–60 mL, whereas Group B received 100–120 mL, representing a substantial reduction in contrast agent usage. This marked contrast reduction is of particular clinical relevance, as the KDIGO 2023 Scope of Work for the updated Acute Kidney Injury guideline addresses strategies for prevention of contrast-associated AKI, highlighting the importance of minimizing contrast exposure to reduce renal complications. Although a 50% reduction in contrast volume is theoretically expected to lower the risk of PC-AKI based on KDIGO guidelines, the present study did not perform a formal comparative analysis of post-contrast renal function changes. Therefore, the clinical benefit in terms of renal safety remains a hypothesis that should be tested in future dedicated studies. Nonetheless, these findings demonstrate that the integrated one-stop protocol incorporating vHP technology not only shortened the net scanning time, using less contrast medium, but also substantially reduced the overall procedural workflow, better reflecting the practical workflow advantages of one-stop imaging in clinical settings.

**Figure 4:**
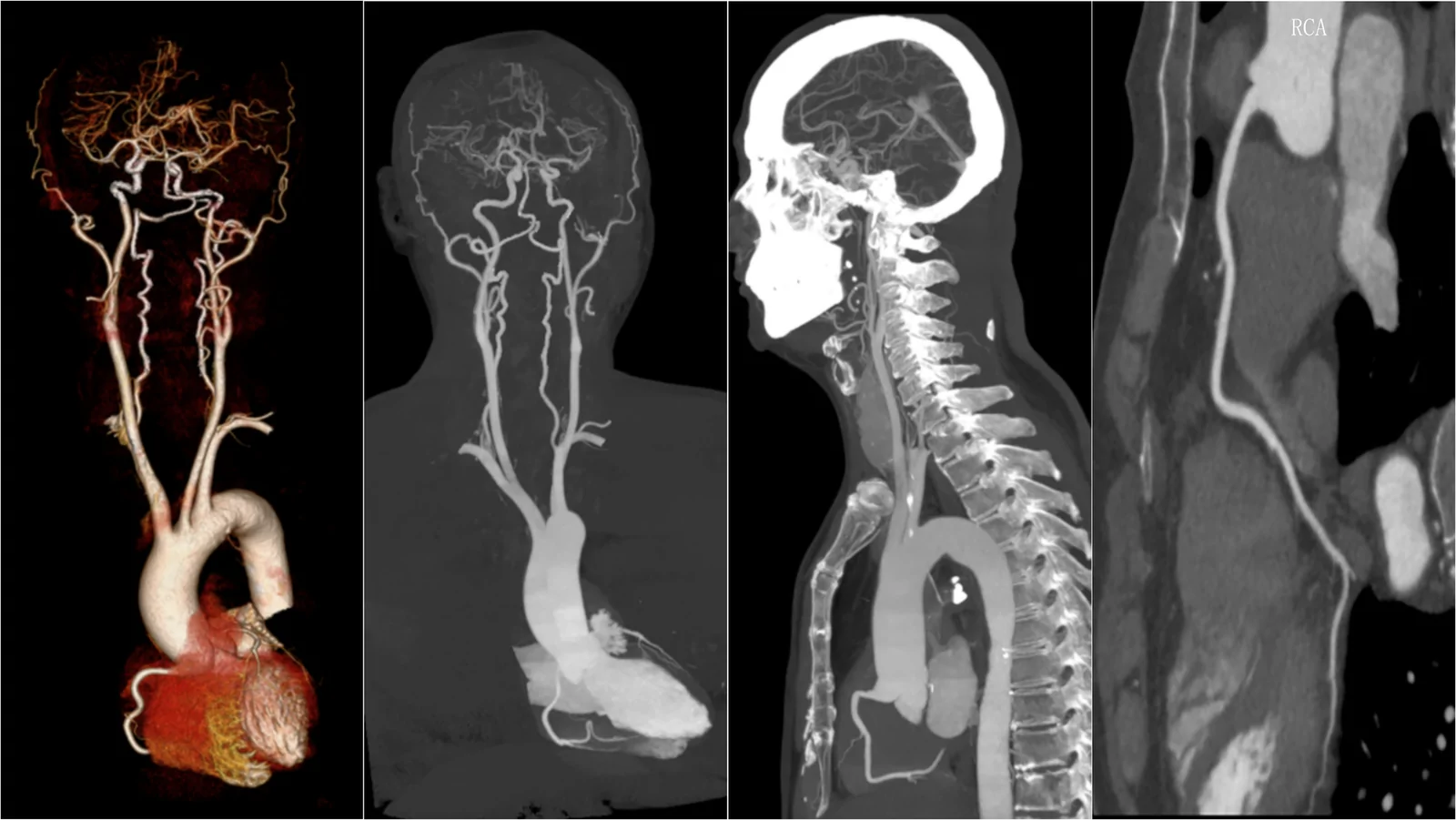
One-stop cardiovascular and cerebrovascular angiography. A 55-year-old male was admitted to the hospital due to chest pain persisting for more than one year. Volume reconstruction, maximum intensity projection (MIP), and surface rendering demonstrate excellent image quality of the head, neck, and coronary arteries, achieving a subjective image quality score of 4 points. The electrocardiogram (ECG) gating trace indicates that the mean heart rate during scanning was 75 beats per minute. The contrast agent volume was 50 mL, the scan duration was 7 seconds, the dose - length product (DLP) was 838 mGy·cm.

**Figure 5:**
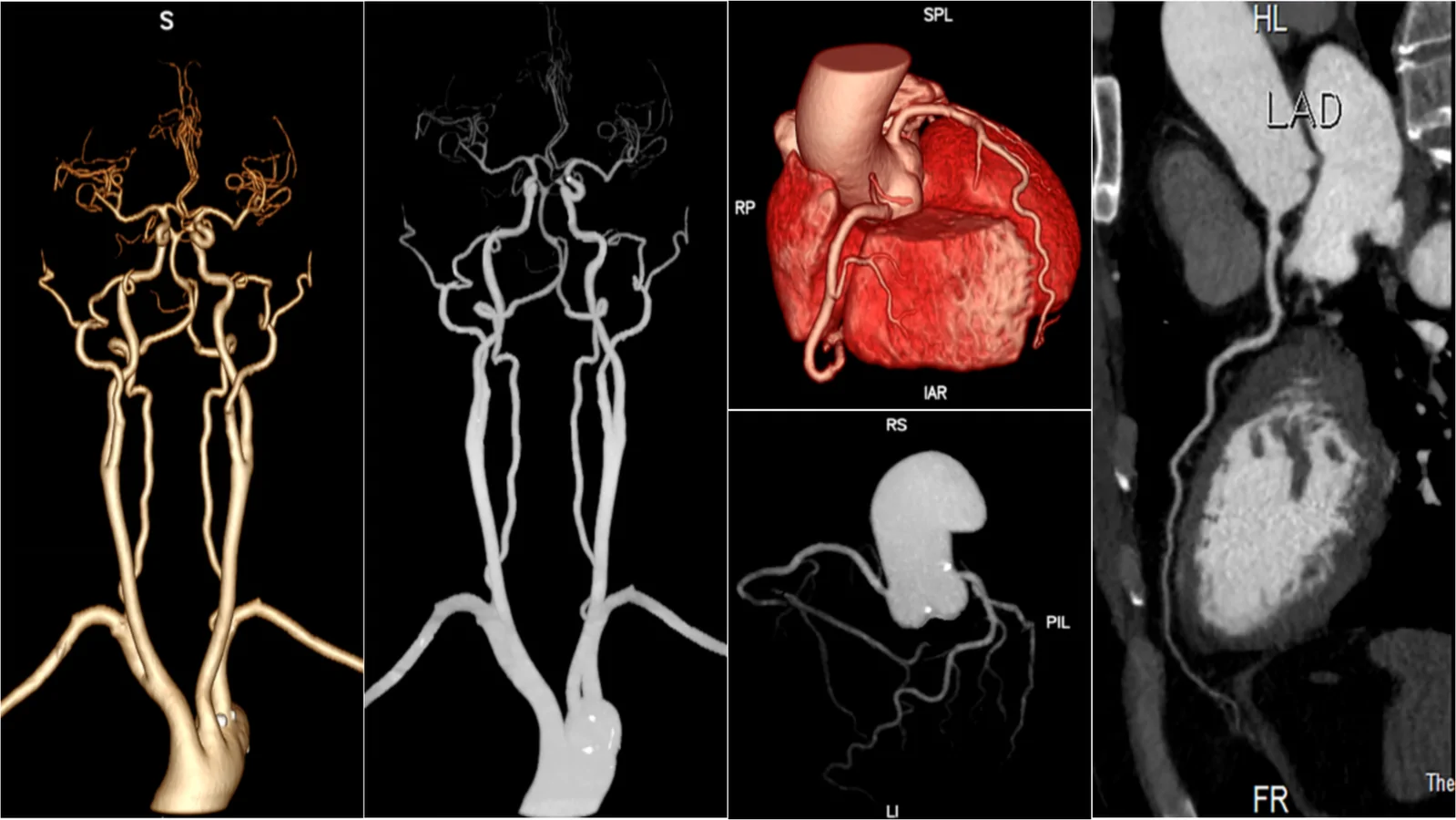
Two imaging tests, pulse CCTA and head and neck CTA. A 57-year-old male admitted to the hospital with a history of hypertension lasting 2 years. Volume reproduction, maximum density projection, and surface reorganization demonstrated excellent image quality of the head, neck, and coronary arteries, yielding a subjective image quality score of 4 points. The electrocardiogram gating diagram indicated an average heart rate of 72 beats per minute during the scanning process. The total contrast agent dosage for both scans was 110 mL, with a cumulative scan duration of 16 seconds. The dose-length product was recorded at 706 mGy·cm.

Notably, the 30.8% higher DLP in the integrated protocol reflects the deliberate use of small-pitch helical acquisition in the cardiac segment to ensure optimal coronary image quality. This dose increase should be interpreted in the context of the substantial workflow benefits achieved: a 57.9% reduction in effective scanning time and a 50% reduction in contrast medium volume. At the same time, the integrated protocol offers clear advantages for coronary and proximal aortic evaluation, but at the cost of higher radiation exposure and relatively higher noise in some intracranial and cervical vessels. The trade-off between radiation dose and these clinical benefits must be considered on a patient-by-patient basis, particularly in younger patients with longer life expectancy. For reference, the DLP values observed in both groups (909.00 mGy·cm for Group A and 694.50 mGy·cm for Group B) are within the range of previously reported values for combined cardiocerebrovascular CT examinations, although specific diagnostic reference levels for this combined indication remain to be established in future multicenter surveys. Therefore, the clinical adoption of the integrated protocol requires careful balancing of the radiation dose penalty against the workflow and contrast-sparing benefits, with patient age and clinical urgency as key modifiers (Figures 4, 5).

Limitations and deficiencies: ① This single-center exploratory study had a small, unbalanced sample (25 vs. 36), which may introduce selection bias. partly due to a higher nondiagnostic image rate in Group A (19.4% vs. 10.0%) reflecting the vHP learning curve. The lack of formal power analysis and the inability to perform heart rate subgroup analyses limit generalizability, particularly for patients with higher heart rates or arrhythmias. Larger multicenter studies are warranted; ② The use of small-pitch helical scanning during coronary CCTA imaging leads to an increased radiation dose within this scanning range; ③ effective radiation doses (ED) were not calculated in this study because segment-specific DLP values could not be retrieved separately from the raw CT data to apply the region-specific k-factors recommended by ICRP Publication 103. Therefore, radiation exposure was compared using total DLP only. Although DLP is a widely accepted surrogate for radiation dose in CT, the absence of ED values limits direct comparison with other studies that report ED. Future studies should prospectively collect segmental DLP data to enable proper ED calculation.④The integrated protocol had a higher nondiagnostic rate than the conventional protocol (19.4% vs. 10.0%), predominantly in older patients. This suggests increased susceptibility to motion artifacts from small-pitch helical acquisition. Operator experience and patient selection may also contribute. The nearly twofold higher exclusion rate implies less robustness in elderly populations—a key group for CTA—and should be weighed against workflow benefits. Further studies with learning curve assessments are needed. ⑤the scanning protocols for Group A and Group B differed in multiple parameters simultaneously (detector collimation, gantry rotation time, pitch, and trigger threshold). Therefore, the observed differences in image quality, radiation dose, and contrast volume cannot be uniquely attributed to the vHP technique alone. This confounding effect represents a fundamental limitation of our study design.⑥Diagnostic accuracy for significant coronary stenosis and plaque morphology was not evaluated due to the absence of an independent reference standard. This study therefore addresses technical feasibility and workflow efficiency, not clinical diagnostic performance. Sensitivity, specificity, and predictive values remain unknown; future validation against invasive angiography is needed before routine use.

## 5 Conclusion

In conclusion, the integrated wide-detector CT protocol evaluated in this study (which incorporates variable helical pitch as a key enabling component) is technically feasible and offers workflow advantages over conventional scanning, including shorter examination time, reduced contrast volume, and preserved subjective and objective image quality, at the cost of a 30.8% higher radiation dose. However, because no independent reference standard (e.g., invasive angiography) was available, its diagnostic accuracy for detecting hemodynamically significant stenosis and characterizing plaque morphology remains unvalidated. Thus, current evidence supports technical feasibility and workflow efficiency, not clinical diagnostic performance, which requires future validation. The observed benefits should be attributed to the combined hardware-software strategy—wider detector, faster rotation, and optimized pitch—rather than to vHP alone.

## Data Availability

The original contributions presented in the study are included in the article/Supplementary Material, further inquiries can be directed to the corresponding authors.

## References

[1] Vaduganathan M Mensah G Turco J. The global burden of cardiovascular diseases and risk: a compass for future health. JACC. (2022) 80(25):2361–71. 10.1016/j.jacc.2022.11.005 36368511

[2] Wu D Hu X Meng L Li J Xu J Zhang L. Influence of loneliness burden on cardio-cerebral vascular disease among the Chinese older adult: a national cohort study. Front Public Health. (2024) 12:1307927. 10.3389/fpubh.2024.1307927 38414893 PMC10896831

[3] Liu Q Zhao J Wang S. From cerebrovascular diseases to neuro-co-cardiological diseases: a challenge in the new epoch. Sci Bull (Beijing). (2022) 67(18):1830–2. 10.1016/j.scib.2022.08.013 36546292

[4] Ogungbe O Longenecker CT Beaton A De Loizaga S Brant LCC Turkson Ocran R-AN. Advancing cardiovascular health equity globally through digital technologies. J Am Heart Assoc. (2024) 13(2):e031237. 10.1161/JAHA.123.031237 38226506 PMC10926780

[5] Muthalaly RG Abrahams TB Nerlekar N Nelson AJ Tan S Chan J. Asymptomatic coronary artery disease in ischaemic stroke survivors: a systematic review and meta-analysis. Eur Stroke J. (2024) 9(3):540–54. 10.1177/23969873241231702 38357886 PMC11418521

[6] Suzuki M Okawa M Okuno Y Yang T Takenobu Y Shiomi H. Prevalence of carotid artery stenosis with coronary artery disease in Japanese patients: a single-center study. J Neurol Sci. (2022) 443:120492. 10.1016/j.jns.2022.120492 36410187

[7] Buckley BJR Harrison SL Hill A Underhill P Lane DA Lip GYH. Stroke-heart syndrome: incidence and clinical outcomes of cardiac complications following stroke. Stroke. (2022) 53(5):1759–63. 10.1161/STROKEAHA.121.037316 35354300

[8] Nakai M Iwanaga Y Sumita Y Wada S Hiramatsu H Iihara K. Associations among cardiovascular and cerebrovascular diseases: analysis of the nationwide claims-based JROAD-DpC dataset. PLoS One. (2022) 17(3):e0264390. 10.1371/journal.pone.0264390 35275919 PMC8916648

[9] Zhao L Bao J Guo Y Li J Yang X Lv T. Ultra-low dose one-step CT angiography for coronary, carotid and cerebral arteries using 128-slice dual-source CT: a feasibility study. Exp Ther Med. (2019) 17(5):4167–75. 10.3892/etm.2019.7420 30988794 PMC6447913

[10] International Commission on Radiological Protection (ICRP). The 2007 recommendations of the international commission on radiological protection. ICRP publication 103. Ann ICRP. (2007) 37(2–4):1–332. 10.1016/j.icrp.2007.10.003 18082557

[11] Abdelkarim A Roy SK Kinninger A Salek A Baranski O Andreini D. Evaluation of image quality for high heart rates for coronary computed tomographic angiography with advancement in CT technology: the CONVERGE registry. J Cardiovasc Dev Dis. (2023) 10(9):404. 10.3390/jcdd10090404 37754833 PMC10532141

[12] Ondrejkovic M Salat D Cambal D Klepanec A. Radiation dose and image quality of CT coronary angiography in patients with high heart rate or irregular heart rhythm using a 16-cm wide detector CT scanner. Medicine (Baltimore). (2022) 101(37):e30583. 10.1097/MD.0000000000030583 36123855 PMC9478320

[13] Fan Y Qin T Sun Q Wang M Liang B. A review of factors affecting radiation dose and image quality in coronary CTA performed with wide-detector CT. Tomography. (2024) 10(11):1730–43. 10.3390/tomography10110127 39590936 PMC11598146

[14] Gulati M Levy PD Mukherjee D Amsterdam E Bhatt DL Birtcher KK. 2021 AHA/ACC/ASE/CHEST/SAEM/SCCT/SCMR guideline for the evaluation and diagnosis of chest pain: a report of the American College of Cardiology/American Heart Association joint committee on clinical practice guidelines. J Am Coll Cardiol. (2021) 78(22):e187–285. 10.1016/j.jacc.2021.07.053 Erratum in: J Am Coll Cardiol. (2024) 84(18):1771. doi: 10.1016/j.jacc.2024.09.024. 34756653

[15] Maroules CD Rybicki FJ Ghoshhajra BB Batlle JC Branch K Chinnaiyan K. 2022 Use of coronary computed tomographic angiography for patients presenting with acute chest pain to the emergency department: an expert consensus document of the society of cardiovascular computed tomography (SCCT): endorsed by the American college of radiology (ACR) and north American society for cardiovascular imaging (NASCI). J Cardiovasc Comput Tomogr. (2023) 17(2):146–63. 10.1016/j.jcct.2022.09.003 36253281

[16] Guo R Deng J Rong P Zhou W Zhang G Peng S. One-stop combined CT angiography of coronary and craniocervical arteries: recommended as the first examination for patients suspected of coronary or craniocervical artery disease. Eur Radiol. (2023) 33(10):7034–43. 10.1007/s00330-023-09528-w [published correction appears in Eur Radiol. (2023) 33(10):7352. doi: 10.1007/s00330-023-09981-7.]. 36905467

[17] Wang Y Chen Y Liu P Lv W Wu J Wei M. Clinical effectiveness of contrast medium injection protocols for 80-kV coronary and craniocervical CT angiography-a prospective multicenter observational study. Eur Radiol. (2022) 32(6):3808–18. 10.1007/s00330-021-08505-5 35103828

[18] Liu S Zhang Z Liu B Zhou S Xie J Han R. One-step integrated coronary-carotid-cerebral computed tomography angiography to evaluate cardiovascular and cerebrovascular atherosclerosis. BMC Cardiovasc Disord. (2023) 23(1):367. 10.1186/s12872-023-03343-3 37480020 PMC10362771

[19] Zhang Q Pan H Ding J Pan J Sun A. The application value of deep learning-based reconstruction combined with segmented contrast injection in “one-stop” cardio-cerebrovascular computed tomography angiography. Quant Imaging Med Surg. (2025) 15(10):9545–58. 10.21037/qims-2024-2579 41081228 PMC12514647

[20] Vañó E Miller DL Martin CJ Rehani MM Kang K Rosenstein M. ICRP Publication 135: diagnostic reference levels in medical imaging. Ann ICRP. (2017) 46(1):1–144. 10.1177/0146645317717209 29065694

[21] Kahraman G Haberal KM Ağıldere AM. Establishment of local diagnostic reference levels for computed tomography with cloud-based automated dose-tracking software in türkiye. Diagn Interv Radiol. (2024) 30(3):205–11. 10.4274/dir.2023.232265 37650522 PMC11095070

[22] Matsumoto S Yamada Y Hashimoto M Okamura T Yamada M Yashima F. CT Imaging before transcatheter aortic valve implantation (TAVI) using variable helical pitch scanning and its diagnostic performance for coronary artery disease. Eur Radiol. (2017) 27(5):1963–70. 10.1007/s00330-016-4547-4 27562479

[23] Li XS Geng JG Zhu YH Liu LY Qiao YQ Ma YL. CT Imaging using variable helical pitch scanning for lower extremity arterial disease: reduced contrast medium dose, improved image quality and diagnostic accuracy. Eur J Radiol. (2024) 181:111792. 10.1016/j.ejrad.2024.111792 Erratum in: Eur J Radiol. (2024) 181:111817. doi: 10.1016/j.ejrad.2024.111817. 39476770

[24] Xing J Zhu L Sun Y. Application and value of vHP technology in “one-stop” CTA scanning of carotid and coronary arteries. J Med Imaging Radiat Sci. (2024) 55(3):101576. 10.1016/j.jmir.2024.101576

[25] Shin N Kim SM Choe YH. Protocol using wide-detector CT with single contrast injection for the aorta and coronary artery: variable helical pitch versus volume scan following helical scan. Int J Cardiovasc Imaging. (2019) 35(10):1935–42. 10.1007/s10554-019-01640-7 31172392

[26] Zhao X Shan Y Zheng C Wang J Zhang M Lu J. Comparison of “one-stop” coronary and head-and-neck computed tomography angiography with different scan sequences using a wide-detector system: heart-to-cranial vs. Cranial-to-heart. Quant Imaging Med Surg. (2025) 15(3):1898–911. 10.21037/qims-24-824 40160655 PMC11948432

[27] Wang M Zheng C Yang L Su J Wang B Sheng J. Low KV-low contrast medium dose one-stop dual source CT high pitch integrated coronary-carotid-cerebral-aortic CTA improves image quality and reduces both radiation and contrast medium doses. Eur J Radiol Open. (2025) 14:100637. 10.1016/j.ejro.2025.100637 39944781 PMC11815692

[28] Sun K Li K Han R Li W Chen N Yang Q. Evaluation of high-pitch dual-source CT angiography for evaluation of coronary and carotid-cerebrovascular arteries. Eur J Radiol. (2015) 84(3):398–406. 10.1016/j.ejrad.2014.11.009 25544556

[29] Liu J Wang C Li Q Duan X Zhu X Wang J. Free-Breathing, non-gated heart-to-brain CTA in acute ischemic stroke: a feasibility study on dual-source CT. Front Neurol. (2022) 13:616964. 10.3389/fneur.2022.616964 35273552 PMC8902348

