# Supplementary material for: A wide-detector CT protocol for one-stop cardio-cerebrovascular angiography: a technical feasibility study comparing an integrated protocol with conventional two-scan approach
Source: Front Radiol. 2026 Sep 1;6:1887780. doi: 10.3389/fradi.2026.1887780 (PMC13575675; doi:10.3389/fradi.2026.1887780)
Supplement: Supplementary file 1 [file Table1.docx]

#### Supplementary Materials

Supplementary Table S1. Baseline characteristics of included versus excluded patients

| Variable | | Included  (n=61) | Excluded  (n-10) | Difference value | Statistic | P value | Effect size |
| --- | --- | --- | --- | --- | --- | --- | --- |
| Age | | 56.57±10.01 | 70.70±7.94 | -14.13 | t=-5.008 | **0.0002** | 1.446 |
| SBP | | 125.70±22.28 | 118.90±12.72 | 6.80 | U=340.0 | 0.5676 | -0.115 |
| DBP | | 82.44±12.34 | 82.90±9.27 | -0.46 | U=283.0 | 0.7214 | 0.072 |
| HR | | 76.08±10.43 | 76.20±8.53 | -0.12 | t=-0.039 | 0.9693 | 0.012 |
| Weight | | 69.93±5.59 | 69.80±3.68 | 0.13 | U=316.0 | 0.8617 | -0.036 |
| Height | | 1.68±0.04 | 1.68±0.04 | -0.00 | U=259.5 | 0.4532 | 0.149 |
| BMI | | 24.83±1.45 | 24.66±0.83 | 0.17 | U=358.5 | 0.3809 | -0.175 |
| Gender | man | 30(49.2%) | 5(50.0%) |  | χ²=0.0 | 1.0 |  |
|  | woman | 31(50.8%) | 5(50.0%) |  |  |  |  |
| Smoking history | no | 30(49.2%) | 6(60.0%) |  | 0.645 | 0.735 |  |
|  | yes | 31(50.8%) | 4(40.0%) |  |  |  |  |
| Hypertension | no | 27(44.3%) | 6(60.0%) |  | 0.529 | 0.4973 |  |
|  | yes | 34(55.7%) | 4(40.0%) |  |  |  |  |
| Diabetes | no | 44(72.1%) | 8(80.0%) |  | 0.647 | 0.7192 |  |
|  | yes | 17(27.9%) | 2(20.0%) |  |  |  |  |
| Hyperlipidemia | no | 40(65.6%) | 6(60.0%) |  | 1.27 | 0.7334 |  |
|  | yes | 21(34.4%) | 4(40.0%) |  |  |  |  |

Continuous variables were compared using Welch's  t-test or Mann–Whitney U test, based on normality (Shapiro–Wilk test). Effect sizes were Cohen's d or rank‑biserial correlation, respectively. Categorical variables were compared using chi‑square or Fisher's exact test. Data are n (%). Two‑sided P < 0.05 was significant.

Supplementary Table S2. ANCOVA results for objective image quality parameters in coronary arteries, aortic branches, cervical vessels, and intracranial arteries, adjusted for age, BMI, heart rate, and systolic blood pressure

|  |  | Group A  LSMean | Group B  LSMean | Difference value | Standard error | 95% CI | F  -value | P  -value | Adj R2 | Resid SD |
| --- | --- | --- | --- | --- | --- | --- | --- | --- | --- | --- |
| RCA | CT value | 531.400 | 536.306 | 4.906 | 6.451 | [-7.739, 17.550] | 0.578 | 0.450035 | -0.0071 | 24.780 |
|  | SD value | 17.888 | 18.489 | 0.601 | 0.267 | [0.077, 1.124] | 5.063 | **0.028188** | 0.0634 | 1.026 |
|  | CNR | 35.153 | 30.259 | -4.894 | 1.125 | [-7.099, -2.688] | 18.916 | **<0.001** | 0.2299 | 4.322 |
|  | SNR | 29.917 | 29.072 | -0.845 | 0.649 | [-2.118, 0.428] | 1.691 | 0.198513 | 0.0114 | 2.495 |
| LAD | CT value | 524.640 | 529.528 | 4.888 | 4.921 | [-4.758, 14.534] | 0.986 | 0.324676 | -0.0002 | 18.903 |
|  | SD value | 18.320 | 18.769 | 0.449 | 0.241 | [-0.023, 0.922] | 3.470 | 0.067478 | 0.0395 | 0.927 |
|  | CNR | 34.641 | 29.828 | -4.813 | 1.121 | [-7.010, -2.617] | 18.453 | **<0.001** | 0.2253 | 4.304 |
|  | SNR | 28.824 | 28.244 | -0.580 | 0.465 | [-1.491, 0.332] | 1.555 | 0.217308 | 0.0092 | 1.786 |
| LCX | CT value | 520.600 | 526.333 | 5.733 | 4.610 | [-3.303, 14.769] | 1.547 | 0.218566 | 0.0090 | 17.709 |
|  | SD value | 18.340 | 18.969 | 0.629 | 0.258 | [0.123, 1.135] | 5.945 | **0.017792** | 0.0761 | 0.992 |
|  | CNR | 34.237 | 29.601 | -4.636 | 1.012 | [-6.620, -2.652] | 20.973 | **<0.001** | 0.2497 | 3.888 |
|  | SNR | 28.492 | 27.697 | -0.794 | 0.437 | [-1.650, 0.062] | 3.309 | 0.073974 | 0.0371 | 1.677 |
| Aorta root | CT value | 544.360 | 539.306 | -5.054 | 4.798 | [-14.459, 4.350] | 1.110 | 0.296431 | 0.0018 | 18.430 |
|  | SD value | 18.360 | 18.139 | -0.221 | 0.192 | [-0.598, 0.156] | 1.323 | 0.254676 | 0.0054 | 0.738 |
|  | CNR | 36.179 | 30.493 | -5.686 | 1.107 | [-7.855, -3.516] | 26.390 | **<0.001** | 0.2973 | 4.251 |
|  | SNR | 30.394 | 28.911 | -1.483 | 0.496 | [-2.455, -0.511] | 8.948 | **0.004050** | 0.1170 | 1.905 |
| Aorta arch | CT value | 548.120 | 536.306 | -11.814 | 6.460 | [-24.476, 0.847] | 3.345 | 0.072473 | 0.0376 | 24.813 |
|  | SD value | 17.728 | 18.072 | 0.344 | 0.208 | [-0.063, 0.752] | 2.739 | 0.103260 | 0.0282 | 0.799 |
|  | CNR | 36.506 | 30.259 | -6.247 | 1.149 | [-8.499, -3.994] | 29.553 | **<0.001** | 0.3224 | 4.414 |
|  | SNR | 31.396 | 28.739 | -2.658 | 0.713 | [-4.055, -1.260] | 13.891 | **<0.001** | 0.1769 | 2.739 |
| LSA | CT value | 524.080 | 518.694 | -5.386 | 3.847 | [-12.925, 2.154] | 1.960 | 0.166748 | 0.0157 | 14.776 |
|  | SD value | 18.776 | 18.386 | -0.390 | 0.276 | [-0.930, 0.150] | 2.000 | 0.162572 | 0.0164 | 1.059 |
|  | CNR | 37.367 | 29.074 | -8.293 | 0.938 | [-10.132, -6.453] | 78.089 | **<0.001** | 0.5623 | 3.605 |
|  | SNR | 28.281 | 28.050 | -0.231 | 0.703 | [-1.610, 1.147] | 0.108 | 0.743533 | -0.0151 | 2.702 |
| LCCA1 | CT value | 526.160 | 513.444 | -12.716 | 5.679 | [-23.846, -1.585] | 5.014 | **0.028931** | 0.0627 | 21.813 |
|  | SD value | 18.748 | 18.969 | 0.221 | 0.297 | [-0.361, 0.804] | 0.556 | 0.459023 | -0.0075 | 1.141 |
|  | CNR | 37.565 | 28.740 | -8.824 | 1.053 | [-10.888, -6.761] | 70.228 | **<0.001** | 0.5357 | 4.045 |
|  | SNR | 28.328 | 27.531 | -0.797 | 0.708 | [-2.184, 0.590] | 1.269 | 0.264569 | 0.0045 | 2.718 |
| BCT | CT value | 523.000 | 512.639 | -10.361 | 4.406 | [-18.998, -1.724] | 5.529 | **0.022065** | 0.0702 | 16.926 |
|  | SD value | 18.324 | 18.703 | 0.379 | 0.241 | [-0.094, 0.851] | 2.467 | 0.121573 | 0.0239 | 0.926 |
|  | CNR | 37.253 | 28.651 | -8.602 | 0.912 | [-10.390, -6.814] | 88.940 | **<0.001** | 0.5944 | 3.504 |
|  | SNR | 28.366 | 26.956 | -1.410 | 0.590 | [-2.566, -0.255] | 5.721 | **0.019967** | 0.0729 | 2.265 |
| LCCA2 | CT value | 517.160 | 507.833 | -9.327 | 5.316 | [-19.746, 1.092] | 3.078 | 0.084535 | 0.0335 | 20.419 |
|  | SD value | 18.340 | 18.689 | 0.349 | 0.230 | [-0.103, 0.801] | 2.293 | 0.135321 | 0.0211 | 0.885 |
|  | CNR | 36.780 | 38.243 | 1.463 | 0.754 | [-0.014, 2.941] | 3.767 | 0.057059 | 0.0441 | 2.896 |
|  | SNR | 29.174 | 26.675 | -2.499 | 0.637 | [-3.749, -1.250] | 15.372 | **<0.001** | 0.1932 | 2.449 |
| RCCA | CT value | 518.040 | 508.722 | -9.318 | 7.196 | [-23.423, 4.787] | 1.677 | 0.200434 | 0.0111 | 27.642 |
|  | SD value | 18.044 | 18.944 | 0.900 | 0.172 | [0.564, 1.237] | 27.537 | **<0.001** | 0.3067 | 0.659 |
|  | CNR | 36.834 | 38.306 | 1.472 | 0.790 | [-0.076, 3.020] | 3.476 | 0.067253 | 0.0396 | 3.033 |
|  | SNR | 28.928 | 26.100 | -2.828 | 0.538 | [-3.883, -1.773] | 27.600 | **<0.001** | 0.3072 | 2.068 |
| LICA | CT value | 506.400 | 498.333 | -8.067 | 5.024 | [-17.915, 1.781] | 2.578 | 0.113730 | 0.0256 | 19.300 |
|  | SD value | 19.280 | 18.969 | -0.311 | 0.262 | [-0.825, 0.204] | 1.402 | 0.241194 | 0.0066 | 1.008 |
|  | CNR | 35.869 | 37.399 | 1.530 | 0.823 | [-0.084, 3.143] | 3.453 | 0.068126 | 0.0393 | 3.162 |
|  | SNR | 26.422 | 26.122 | -0.300 | 0.500 | [-1.280, 0.679] | 0.361 | 0.550335 | -0.0108 | 1.919 |
| RICA | CT value | 504.920 | 521.722 | 16.802 | 4.420 | [8.140, 25.465] | 14.453 | **<0.001** | 0.1832 | 16.976 |
|  | SD value | 19.228 | 18.489 | -0.739 | 0.189 | [-1.109, -0.369] | 15.357 | **<0.001** | 0.1931 | 0.724 |
|  | CNR | 35.722 | 39.525 | 3.804 | 0.777 | [2.281, 5.326] | 23.983 | **<0.001** | 0.2770 | 2.983 |
|  | SNR | 25.896 | 25.706 | -0.190 | 0.525 | [-1.219, 0.838] | 0.132 | 0.717924 | -0.0147 | 2.015 |
| LVA | CT value | 487.240 | 482.028 | -5.212 | 4.993 | [-14.998, 4.574] | 1.090 | 0.300767 | 0.0015 | 19.178 |
|  | SD value | 19.756 | 18.969 | -0.787 | 0.257 | [-1.290, -0.283] | 9.383 | **0.003298** | 0.1226 | 0.986 |
|  | CNR | 34.228 | 35.917 | 1.689 | 0.852 | [0.020, 3.359] | 3.935 | 0.051948 | 0.0466 | 3.271 |
|  | SNR | 24.737 | 25.281 | 0.544 | 0.447 | [-0.332, 1.420] | 1.480 | 0.228631 | 0.0079 | 1.717 |
| RVA | CT value | 486.120 | 474.194 | -11.926 | 6.013 | [-23.711, -0.140] | 3.933 | 0.051997 | 0.0466 | 23.097 |
|  | SD value | 19.516 | 19.139 | -0.377 | 0.225 | [-0.817, 0.063] | 2.821 | 0.098311 | 0.0295 | 0.862 |
|  | CNR | 34.114 | 35.205 | 1.090 | 0.886 | [-0.646, 2.827] | 1.515 | 0.223312 | 0.0085 | 3.403 |
|  | SNR | 24.569 | 24.806 | 0.236 | 0.480 | [-0.705, 1.177] | 0.242 | 0.624324 | -0.0128 | 1.844 |
| LMCA M1 | CT value | 461.080 | 463.528 | 2.448 | 4.935 | [-7.224, 12.120] | 0.246 | 0.621707 | -0.0127 | 18.954 |
|  | SD value | 20.180 | 19.669 | -0.511 | 0.182 | [-0.867, -0.154] | 7.874 | **0.006783** | 0.1028 | 0.699 |
|  | SNR | 22.864 | 23.594 | 0.730 | 0.369 | [0.007, 1.454] | 3.911 | 0.052642 | 0.0463 | 1.419 |
| RMCA M1 | CT value | 453.920 | 455.611 | 1.691 | 5.321 | [-8.738, 12.120] | 0.101 | 0.751746 | -0.0152 | 20.439 |
|  | SD value | 20.464 | 20.028 | -0.436 | 0.240 | [-0.906, 0.034] | 3.312 | 0.073839 | 0.0371 | 0.921 |
|  | SNR | 22.267 | 22.800 | 0.533 | 0.434 | [-0.316, 1.383] | 1.513 | 0.223601 | 0.0085 | 1.665 |
| BA | CT value | 436.080 | 439.500 | 3.420 | 4.974 | [-6.329, 13.169] | 0.473 | 0.494417 | -0.0089 | 19.106 |
|  | SD value | 22.832 | 22.197 | -0.635 | 0.188 | [-1.003, -0.267] | 11.446 | **0.001278** | 0.1483 | 0.721 |
|  | SNR | 19.122 | 19.844 | 0.723 | 0.267 | [0.200, 1.245] | 7.349 | **0.008776** | 0.0957 | 1.024 |

LSMean, least‑squares mean; CI, confidence interval; Adj R², adjusted R‑squared. All models adjusted for age, BMI, HR, SBP, and DBP. Adj R² indicates the proportion of variance explained by the model.
